# Ultrashort Peptides and Gold Nanoparticles: Influence of Constrained Amino Acids on Colloidal Stability

**DOI:** 10.3389/fchem.2021.736519

**Published:** 2021-10-01

**Authors:** Silvia Locarno, Raffaella Bucci, Elisa Impresari, Maria Luisa Gelmi, Sara Pellegrino, Francesca Clerici

**Affiliations:** ^1^ Dipartimento di Fisica “Aldo Pontremoli”, Università degli Studi di Milano, Milano, Italy; ^2^ DISFARM-Dipartimento di Scienze Farmaceutiche, Sezione Chimica Generale e Organica ‘‘A. Marchesini”, Università degli Studi di Milano, Milano, Italy

**Keywords:** ultrashort peptides, biotin, self-assembly, constrained amino acids, gold nanoparticle

## Abstract

Poor colloidal stability of gold nanoparticles (AuNPs) in physiological environments remains one of the major limitations that contribute to their difficult translation from bench to clinic. For this reason, an active research field is the development of molecules able to hamper AuNPs aggregation tendency in physiological environments. In this context, synthetic peptides are gaining an increased interest as an alternative to the use of biomacromolecules and polymers, due to their easiness of synthesis and their profitable pharmacokinetic profile. In this work, we reported on the use of ultrashort peptides containing conformationally constrained amino acids (AAs) for the stabilization of AuNPs. A small library of non-natural self-assembled oligopeptides were synthesized and used to functionalize spherical AuNPs of 20 nm diameter, *via* the ligand exchange method. The aim was to investigate the role of the constrained AA, the anchor point (at C- or N-*terminus*) and the peptide length on their potential use as gold binding motif. Ultrashort Aib containing peptides were identified as effective tools for AuNPs colloidal stabilization. Furthermore, peptide coated AuNPs were found to be storable as powders without losing the stabilization properties once re-dispersed in water. Finally, the possibility to exploit the developed systems for binding proteins *via* molecular recognition was also evaluated using biotin as model.

## Introduction

Metallic nanoparticles (NPs) have been widely exploited in various industrial applications, due to their intrinsically different physical and chemical properties with respect to bulk metals (e.g., higher specific surface areas, specific optical properties, mechanical strengths). NPs peculiar properties mostly arise from their large surface-area-to-volume ratio and the spatial confinement of electrons and electric fields in and around these particles (Sau et al., 2010). In this context, gold NPs (AuNPs) have been particularly investigated (Thompson, 2007), demonstrating to be powerful and versatile tools (Chen et al., 2008; Egorova et al., 2020). AuNPs own indeed the same dimensional scale of biological molecules, making them interesting candidates for biomedical applications as biosensors (Jakhmola et al., 2012), imaging agents (Haun et al., 2010), selective targeting (Avvakumova et al., 2016), drug carriers (Song et al., 2012), and a valid alternative to platinum-based anticancer coordination compounds (Rimoldi et al., 2018; Facchetti and Rimoldi, 2019) for targeted cancer therapy (Yang and Chithrani, 2015).

One of the major limitations of AuNPs is the poor colloidal stability in physiological environments that contributes to poor translation of NPs formulations from bench to clinic. The high presence of proteins and salts in serum can indeed favor NPs aggregation affecting the fate and the interaction capability of AuNPs with biological systems. The stabilization of AuNPs with capping agents is required to enable any biological applications (Foo et al., 2017), although commonly used stabilizing agents, such as citrate and cetyltrimethylammonium bromide (CTAB), are not stable in several buffers. Therefore, an active field of research is the development of molecules able to stabilize AuNPs in the physiological environment. In this context, synthetic peptides are gaining an increased interest as an alternative to the use of biomacromolecules and polymers for the AuNPs functionalization, due to their easiness of synthesis and to the optimal pharmacokinetic profiles, easily tuned by tailoring the peptide sequence. One example is the CALNN motif, designed by Levy (Levy et al., 2004). CALNN decorated AuNPs are particularly stable but the *in vivo* utility is hampered by proteases sensitivity. The introduction of non-natural or non-proteinogenic amino acids (AAs) could be a strategy to overcome this issue (Parween et al., 2013; Bucci et al., 2017; Contini et al., 2018) representing also a versatile approach to reduce peptide conformational flexibility by inducing a well-defined secondary structure (Aschi et al., 2003; Schade et al., 2010; Bucci et al., 2019; Oliva et al., 2019; Vaghi et al., 2020; Bucci et al., 2021). C^α^-tetrasubstituted AAs represent a wide class of non-proteinogenic compounds with well recognized folding induction ability (Toniolo et al., 2002), beingα-amino isobutyric acid (Aib) known as a strong promoter of helical structure (Crisma and Toniolo, 2015).

Peptides containing Alanine and Aib residues have been already used for the functionalization of AuNPs. Schade at al. functionalized very small AuNPs (1–2 nm) with the two octapeptides Trt-S-(CH_2_)_2_-O-CO-Aib-Ala-(Aib)_6_-OMe and Trt-S-(CH_2_)_2_-O-CO-(Aib)_3_-Ala-(Aib)_4_-OMe labelled on Ala with ^13^C = O isotope for using them as local thermometers (Schade et al., 2010). Longo et al. passivated 2 nm AuNPs with Ala-Aib oligopeptides to induce chiroptical properties in nanoparticles (Longo et al., 2013). However, the use of ultrashort peptides containing cyclic or acyclic constrained C^α^-tetrasubstituted AAs with the purpose of increasing AuNPs colloidal stability in physiological environment has not been reported so far.

In our group, we studied the self-assembly propensity of helical Ac-Ala-X-Ala-Aib-Ala-NH_2_ [ X = Aib or cyclopentane amino acid (Ac5c)] peptide sequences that were found to be able to form micelle-likes aggregates capable to encapsulate hydrophobic small molecules such as curcumin (Locarno et al., 2020).

Here, we exploited the possibility to use similar ultrashort peptides as a tool for the colloidal stabilization of AuNPs. We synthesized a small library of peptides containing hydrophobic Alanine and Aib or Ac5c AAs, together with cysteine or thiopropionic acid as gold binding moiety. The choice of these sequences derived also from the properties of these AAs to induce a helical structure. It is indeed known the importance of this type of secondary structure for the stabilization of AuNPs (Schade et al., 2010). Our aim was to investigate the role of the constrained AA, the anchor point (at C- or N-terminus) and the peptide length on their potential use as gold binding motif. The colloidal stability of the so functionalized AuNPs was investigated by UV-Vis spectroscopy, DLS and TEM. Finally, the conjugation of protein by means of specific molecular recognition was evaluated. To this aim, we functionalized the ultrashort peptides with biotin evaluating AuNPs ability to bind avidin.

## Materials and Methods

All chemicals were purchased from Sigma-Aldrich, Iris Biotech or Fluka. All solvents were of ACS grade or higher and were obtained from Sigma-Aldrich. Human AB Serum, male only, off the clot, heat inactivated (HS) was purchased from Seralab.

Peptide purification was performed by semi-preparative RP-HPLC using a DENALI C18 column (10 μm, 250 × 22 mm). Purity was analyzed by analytical HPLC VWR Hitachi (Elite LaChrom) using an Eclipse XDB_C18 column, 5µm, (4.6 × 150 mm). NMR spectroscopic experiments were carried out on 300 MHz spectrometer (300 and 75 MHz for ^1^H and ^13^C, respectively). NMR spectroscopic experiments were carried out on 400 MHz spectrometer (400 and 100 MHz for ^1^H and ^13^C, respectively). Chemical shifts are given in ppm relative to solvent used as internal standard and coupling constants J are reported in hertz (Hz). Mass spectra were acquired on Fisons MD800 spectrometer and electrospray ion trap on a Finnigan LCQ advantage Thermo-spectrometer. FT-IR spectra were recorded on Biodiesel FT-IR FAME Analyzer, composed of a Frontier™ ATR FT-IR spectrometer. Spectra were acquired from 4,000 to 500 cm^−1^ at a resolution of 4 cm^−1^ with 120 scans per sample. Absorption spectra were carried on Agilent Technologies, cary 100 UV-vis spectrophotometer using a cuvette of path 1 cm, in the spectral region 400–800 nm at 25°C. The loading of peptides on AuNPs was measured by semi-preparative RP-HPLC using a LUNA 5 μ C-8. The Dynamic Light Scattering (DLS) measurements were performed in low volume disposable cuvettes using a Malvern Zetasizer Nano ZS90 instrument at 25°C, equipped with 633 nm solid state He–Ne laser at a scattering angle of 90°. Analyses were performed in water (viscosity: 0.8872 Cp, refractive index: 1.33). The size measurements were averaged from at least three repeated measurements. Transmission electron microscope (TEM) analysis were performed on L120C (ThermoFisher, United States) operating at 120 kV; images were acquired by a Ceta camera 4kx4k.

### Synthesis and Characterization of Peptides

The peptides (Table 1) were prepared by solid phase peptide synthesis (SPPS) using the Fmoc/t-Bu strategy. All the peptides were synthesized manually on Rink-Amide resin (0.217 g, loading 0.69 mmol/g), according to the procedure described in the SI. The coupling systems used was 3:5:5:8 equivalents of Fmoc-amino acid/HOBt/HBTU/DIEA for the first two amino acids and for the third ones of peptides 1, 4, 6, 9 and 10, whereas the other couplings were performed using 3:5:5 equivalents of Fmoc-amino acid/HBTU/DIC. The coupling with biotin was performed directly on resin (see SI), using as coupling system 10:10:10 equivalents of biotin/DIC/Oxyma Pure. After each coupling, a Kaiser test was performed to check the completeness of the reaction. The peptidyl bound resins were cleaved with a mixture of TFA/phenol/H_2_O/thioanisole/TIPS (84:5:5:5:1 v/v/v/v) for 2 h under magnetic stirring. After the work-up, the crude peptides were purified by semi-preparative RP-HPLC (see SI for the details) and lyophilized. Finally, the pure peptides were analyzed by ESI-MS, RP-HPLC, ^1^H and ^13^C-NMR spectroscopy.

**TABLE 1:** Synthesized peptides. The gold anchor point is indicated in red.

Compound	Sequence	
**1**	Ac-Ala-Aib-Ala-Aib-A-Cys-NH_2_	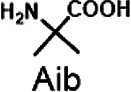
**2**	Ac-Cys-Ala-Aib-Ala-Aib-Ala-NH_2_	
**3**	HS-CH_2_CH_2_CO-Ala-Aib-Ala-Aib-Ala-NH_2_	
**4**	Ac-Ala-Aib-Ala-Cys-NH_2_	
**5**	HS-CH_2_CH_2_CO-Ala-Aib-Ala-NH_2_	
**6**	Ac-Ala-Ac5c-Ala-Aib-Ala-Cys-NH_2_	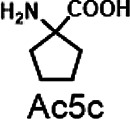
**7**	HS-CH_2_CH_2_CO-Ala-Ac5c-Ala-Aib-Ala-NH_2_	
**8**	HS-CH_2_CH_2_CO-A-Ac5c-A-NH_2_	
**9**	Biotin-Ala-Aib-Ala-Aib-Ala-Cys-NH_2_	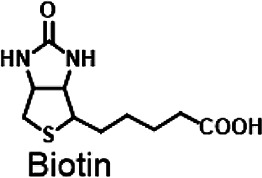
**10**	Biotin-Ala-Ac5c-Ala-Aib-Ala-C-NH_2_	

### Gold Nanoparticles Synthesis

AuNPs with 20 nm diameter are prepared by a modified Turkevich-Frens method (Turkevich et al., 1951; Giljohann et al., 2010). A boiling solution of HAuCl_4_ trihydrate (15 mg, 40 μmol) in 150 ml of Milli-Q water is quickly mixed with 4.5 ml of warm (60–80°C) 1% (wt) aqueous solution of trisodium citrate, followed by reflux for 60 min. The resulting nanoparticle suspension was then allowed to cool down to room temperature and stirred overnight.

The concentration *c* of AuNPs in solution (0.9 nM) was calculated from absorption (A) at 450 nm for a standard path length *l* of 1 cm according to Lambert-Beer Law (Eq.2.1).

$$c=A_{\mathrm{450/}\varepsilon \mathrm{450}}\cdot l$$ (2.1)

ε_450_ is the molar extinction coefficient of AuNPs at 450 nm in water and it depends on the size of nanoparticles. The theoretical value for AuNPs with diameter of 20 nm is 5.41 × 10^8^ Mcm according to the literature data (Haiss et al., 2007). From the concentration, it is possible to calculate the number of particles in solution. All experiments were performed on 2 ml of AuNPs dispersion (***V***), which contains 1.08 × 10^13^ particles according to the following equation:

$$N_{\mathrm{AuNPs}}=c.N_{AV}\cdot V$$ (2.2)

where ***N***_***AV***_ is Avagadro constant (6.02 × 10^23^ mol^−1^).

### Gold Nanoparticles Functionalization

The functionalization of AuNPs with peptides is carried out following a procedure described by (Levy et al., 2004).

All peptides were dissolved in DMSO to give 0.5 mM stock solution. Since 2 ml of AuNPs solution contains 1.08 × 10^13^ nanoparticles, 3.5 × 10^16^ peptide molecules (N_p_) (117 μl of 0.5 mM solution) were calculated to exchange all citrate ions and completely cover nanoparticles. An excess of peptide (175 μl, 1.5-fold excess) has been used to facilitate the exchange reaction and to be sure to create the self-assembled monolayer.

For the AuNPs functionalization, the proper peptide was added to 2 ml of 0.9 nM AuNPs solution under vigorous stirring, and the mixture was left under stirring overnight. Then peptide and citrate excess was washed out by centrifugation at 14,000 rpm for 15 min.

### Stability Studies in Physiological Environment

AuNPs stability was studied by UV-vis spectroscopy and DLS by incubating nanoparticles in different environments: water, phosphate buffer saline (PBS, pH 7.4), Human Serum (HS) 10% in water or HS 100%. An aliquot of concentrated nanoparticles was dispersed in the choosen medium and the stability was evaluated over the time. In the case of 100% HS, an aliquot of concentrated nanoparticles was dispersed in the medium and incubated for 24 h at 37°C, followed by centrifugation at 14,000 rpm for 15 min and redespersion in water. Subsequently, the absorption spectra of nanoparticles in the 400–700 nm range were measured.

### Avidin Recognition

An aqueous solution of avidin (0.125 mg/ml or 0.0125 mg/ml, 20 μL) was added to 2 ml (0.9 nM) of biotinylated peptides AuNPs suspension. The reaction mixture was mixed thoroughly for 2 h at room temperature. Subsequently, functionalized AuNPs were washed by centrifugation (14,000 rpm for 15 min) and the supernatant was analyzed by BCA protein assay (Sigma Aldrich) and HABA test.

### Gel Electrophoresis

Agarose gel electrophoresis of AuNPs was carried out using a Biorad electrophoresis gel apparatus. Small aliquots (16 μl) of the conjugates were supplemented with 30% glycerol (4 μL) and loaded on 0.75% agarose gel in 1X Tris-acetic acid-EDTA (TAE) buffer, and ran for 1–2 h at 80 V. After migration, nanoparticles were visible with the unaided eye as a red band.

## Results and Discussion

### Rational Design and Synthesis of Peptides

The rational design of the peptides considered the need of a strong affinity for gold and a good balance between hydrophobicity and hydrophilicity to ensure both self-assembly and colloidal stabilization. To this purpose, Ala-Aib containing sequences seemed particularly profitable. Indeed, they possess helical conformation, can form stable self-assemblies in water (Locarno et al., 2020), and can be easily functionalized with a thiol group allowing gold binding. We thus prepared a small peptide library (Table 1) by modulating: 1) the sulphur anchor point (at C- or N-*terminus*, using respectively Fmoc-Cys (Trt)-OH or 3-(Tritylthio)propionic acid); 2) the peptide length; 3) the amino acid composition. In this last case, we investigated the effect of Ac5c insertion. Ac5c is a more constrained C^α^-tetrasubstituted AA with respect to Aib that, although its steric hindrance, it seems not decreasing the peptide self-assembly behavior (Locarno et al., 2020). Finally, peptides sequence one and six were functionalized with biotin at N-*terminus* allowing avidin protein binding *via* molecular recognition*.*

All the peptides (Table 1) were prepared by solid phase peptide synthesis (SPPS) (see SI for the details) using the Fmoc/t-Bu strategyon Rink-Amide resin (0.217 g, loading 0.69 mmol/g). Different coupling systems (HOBt/HBTU and DIPEA oror HOBt/DIC or DIC/Oxyma Pure) were used depending on the type, the position and the steric hinderance offered by the single functionality (natural AA, Cα-tetrasubstituted AA, thiol gold binding moiety and biotin). The purity of the synthetized peptides was calculated by analytical RP-HPLC, while their identity was confirmed by NMR and mass spectrometry Helical propensity was finally assessed by FT-IR on selected peptides (see SI).

### Gold Nanoparticles Synthesis and Functionalization with the Peptides.

**FIGURE 1:**
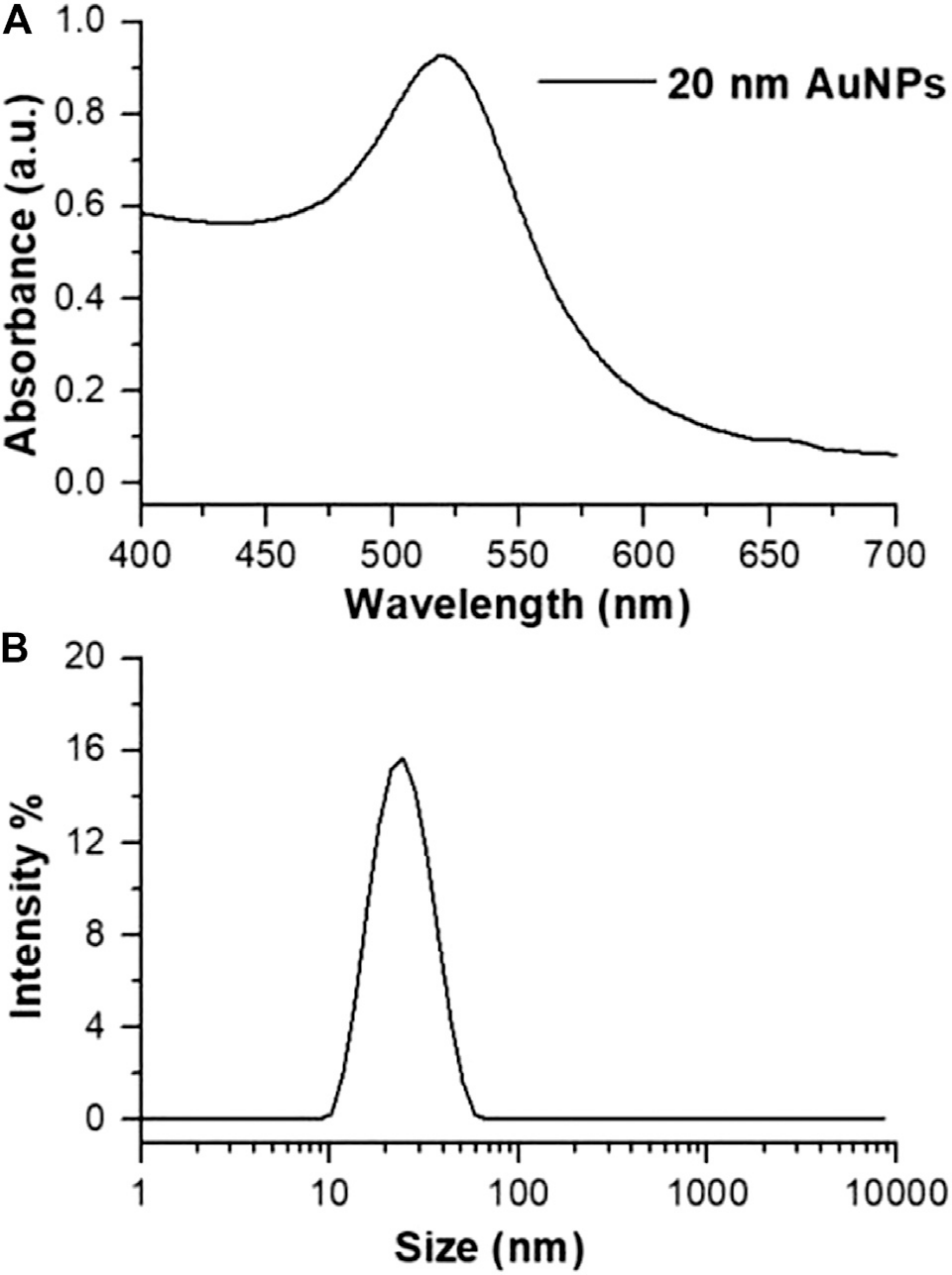
**(A)** Absorption spectrum and **(B)** size distribution by intensity of synthesized citrate@AuNPs in water.

Citrate-stabilized AuNPs were prepared by modified Turkevich-Frens method (Turkevich et al., 1951; Giljohann et al., 2010) and were characterized by UV-spectroscopy. The absorption spectrum in water of synthesized AuNPs are reported in Figure 1A. SPR peak occurred at 518 nm and the calculated A_spr_/A_450_ value is 1.73 which corresponds to an average size of 20 nm (Haiss et al., 2007; Rahman, 2016). This value was also confirmed by DLS (Figure 1B, 22.7 ± 0.2 nm, PdI 0.2 ± 0.02).

The most common approach to functionalize AuNPs is the ligand exchange method involving the displacement of one ligand for another. In the presence of molecules bearing a thiol group, citrate-capped AuNPs undergo ligand exchange giving functionalized nanoparticles (Zong et al., 2017).

In order to establish the amount of peptide needed for the functionalization, the circular peptide axial footprint, which is defined as the average area each peptide occupies on the nanoparticle surface, was measured. The peptide area (A_p_ = 0.38 nm^2^) has been approximated according to Eq. 3.1, measuring the peptide axial average diameter 4) from the crystal structure of the pentapeptide analogue Ac-Ala-Aib-Ala-Aib-Ala-NH_2_, which has been found of 7 Å (Locarno et al., 2020):

$$A_{p}=\pi {\left(d/2\right)}^{2}$$ (3.1)

The ratio between the AuNPs and the peptide areas (K = A_AuNPs_/A_p_) gives the average number of peptides per particle with a 20 nm radius. Some assumptions regarding surface area were made: first, nanoparticles were modelled as a perfect sphere; second, the numbers of particles larger and smaller than the average particle size were assumed to be equal; third, peptides were assumed to be evenly distributed on the nanoparticle surface. According to these assumptions, K is 3,265 peptides/AuNP. Conversely, in the ligand exchange experiments we used 175ul of a 0.5 mM peptide DMSO solution (see *Gold Nanoparticles Functionalization*).

**FIGURE 2:**
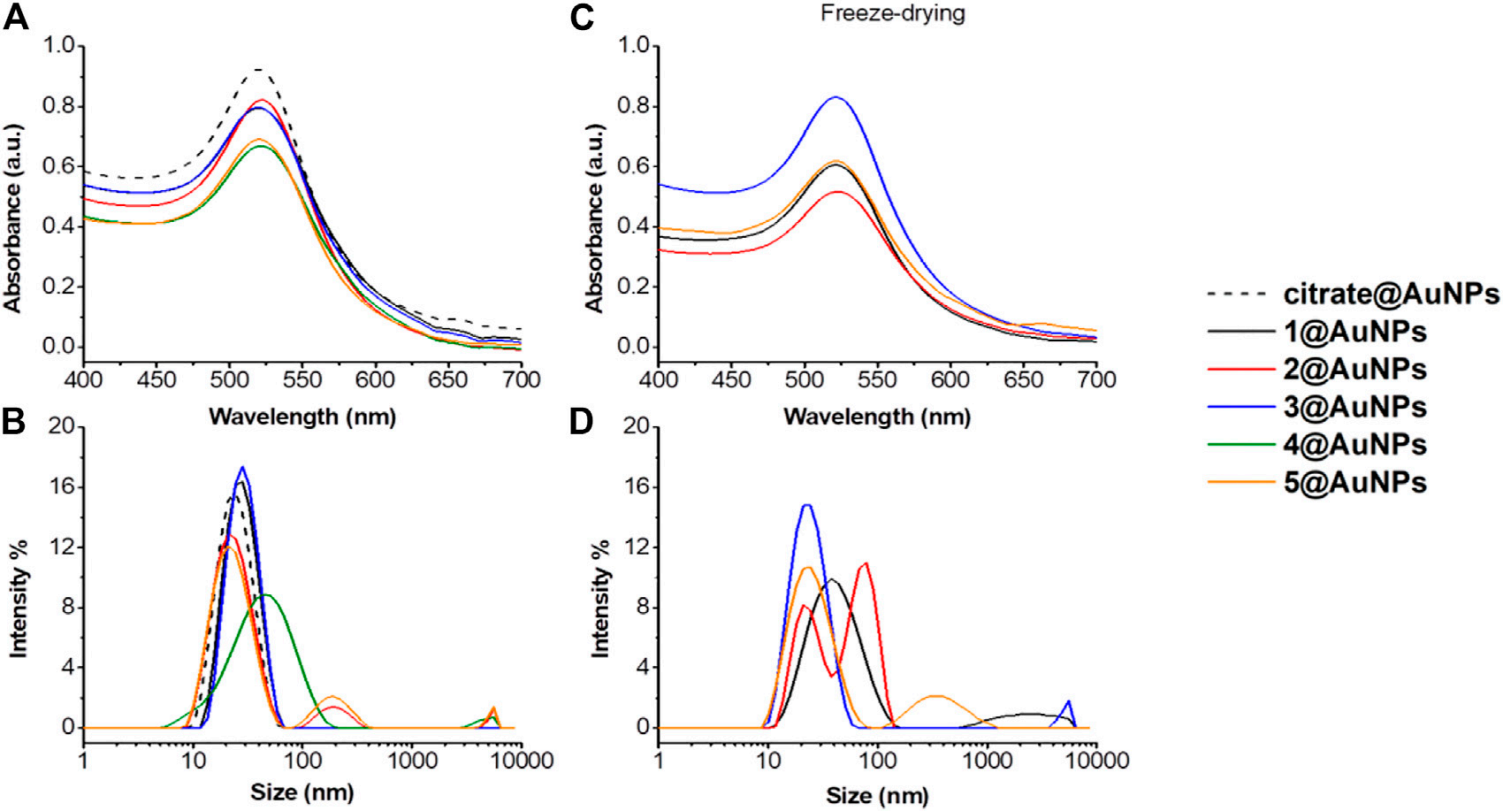
**(A)** Absorption spectra and **(B**) size distribution by intensity of citrate@AuNPs (dash) and functionalized-AuNPs with peptides **1** (black), **2** (red), **3** (blue), **4** (green) and **5** (orange). Mean DLS results are given for three different measurements. The hydrodynamic diameter is found 22.7 ± 0.2 nm PdI 0.2 ± 0.02 for citrate@AuNPs, 31.8 ± 1.1 nm PdI 0.2 ± 0.03 for **1**@AuNPs, 29.1 ± 1.0 nm PdI 0.4 ± 0.02 for **2**@AuNPs, 26.3 ± 1.9 nm PdI 0.2 ± 0.01 for **3**@AuNPs, 69.2 ± 8 nm PdI 0.4 ± 0.1 for **4**@AuNPs, and 27.1 ± 1.8 nm PdI 0.3 ± 0.008 for **5**@AuNPs. **(C)** Absorption spectra and **(D)** size distribution by intensity of functionalized-AuNPs with peptides **1** (black), **2** (red), **3** (blue) and **5** (orange) after freeze-drying and subsequent water dispersion. Mean DLS results are given for three different measurements. The hydrodynamic diameter is found 42.4 ± 3.8 nm PdI 0.3 ± 0.07 for **1**@AuNPs, 19.6 ± 0.3 and 90.4 ± 3.2 nm PdI 0.2 ± 0.03 for **2**@AuNPs, 24.7 ± 0.6 nm PdI 0.3 ± 0.02 for **3**@AuNPs, and 26.3 ± 0.8 nm PdI 0.4 ± 0.03 for **5**@AuNPs.

The effect of peptides functionalization on AuNPs was evaluated by UV-Vis and DLS. The functionalization of AuNPs modifies the position and width of SPR absorption since this reaction changes the chemical and physical environment of the particle and consequently, its optical properties. The monitoring of the SPR signal is considered an easy method to control the functionalization reaction and the stability of AuNPs (Krpetic et al., 2011). In the case of Ala-Aib peptides **1-5**, the redshift of UV−Vis plasmon absorption from 518 to 525 nm, is the first confirmation of the AuNPs functionalization (Figure 2A). Moreover, changes in size-distribution features of nanoparticles determined by DLS were observed (increased diameters from 22 to 26–30 nm, Figure 2B). Only in the case of tripeptide **four** aggregation was observed (Figure 2B). This is a consequence of the presence of Cysteine at C-terminus. In fact, as demonstrated below, functionalization of AuNPs by peptides with Cysteine instead of thiopropionic acid, gives less stable AuNPs. Figure 2 shows also the effect of peptide length on AuNPs stability. For peptide **4**, which is the shortest peptide, the aggregation parameters increased rapidly, as shown in Figure 2A,B As the length of the peptide increased, the aggregation was less evident, suggesting a direct correlation between peptide length and stability of the peptide-capped AuNPs. The colloidal stability of capped AuNPs after freeze-drying was then studied to evaluate the possibility to store them as a powder. This is an important parameter since very few AuNPs preparations beside CALNN-capped AuNPs can be stored in dry state and redispersed in water (Levy et al., 2004). As shown in Figure 2 C-D, only **3**@AuNPs maintained the same hydrodynamic diameter after redispersion in water, while for **5**@AuNPs aggregation started to occur. However, aggregation was more evident in case of **1**@AuNPs and **2**@AuNPs, both containing Cysteine residue. These data suggested that in freeze-drying process, the gold anchor point plays a more crucial role than the peptide length.

Taken together all the data we can thus postulate that pentapeptides and thiopropionic acid are the best combination for inhibiting AuNPs aggregation.

**FIGURE 3:**
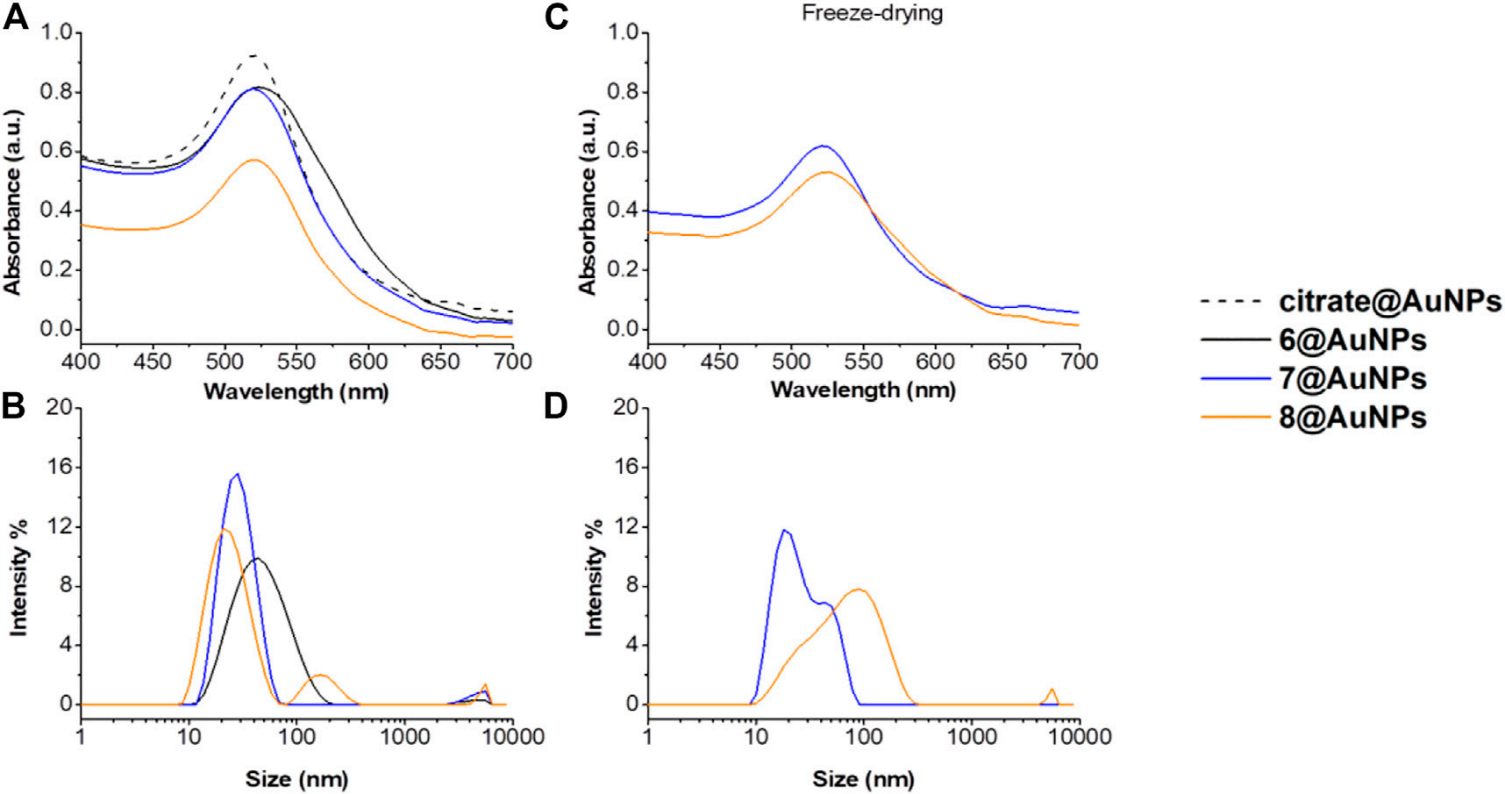
**(A)** Absorption spectra and **(B)** size distribution by intensity of citrate@AuNPs (dash) and functionalized-AuNPs with peptides **6** (black), **7** (blue), **8** (orange). Mean DLS results are given for three different measurements. The hydrodynamic diameter is found 22.7 ± 0.2 nm PdI 0.2 ± 0.02 for citrate@AuNPs, 52.1 ± 4.5 nm PdI 0.3 ± 0.04 for **6**@AuNPs, 27.4 ± 0.3 nm PdI 0.2 ± 0.03 for **7**@AuNPs, and 29.2 ± 1.6 nm PdI 0.3 ± 0.02 for **8**@AuNPs. **(C)** Absorption spectra and **(D)** size distribution by intensity functionalized-AuNPs with peptides **7** (blue) and **8** (orange) after freeze-drying. Mean DLS results are given for three different measurements. The hydrodynamic diameter is found 22.4 ± 2.1 nm PdI 0.2 ± 0.04 for **7**@AuNPs, and 82.7 ± 3.6 nm PdI 0.3 ± 0.03 for **8**@AuNPs.

Peptides **6**, **7** and **8** containing the hindered Ac5c AA were then evaluated by UV-Vis and DLS (Figure 3). Also in this case, when Cysteine is at C-*terminus* (peptide **6**), AuNPs showed evident sign of aggregation: in addition to the color change of suspension, the SPR peak resulted to be broader. Moreover, the hydrodynamic diameter increased more than 20 nm (Figures 3A,B). Peptides **7** and **8**, instead, were able to stabilize the particles. However, after freeze-drying **8**@AuNPs were completely destabilized and big aggregates were observed in DLS profiles (Figure 3D). On the other hand, 7@AuNPs were found more stable, although a partial aggregation started to occur, since a small second population was observed in DLS experiments (Figures 3C,D). These results suggested that the steric hindrance of Ac5c could have negative influence on the AuNPs stability.

### Gold NPs Functionalization With Biotinylated Peptides

From UV-Vis and DLS data, peptides **3** and **7**, both containing thiopropionic acid at *N*-*t*erminus*,* represented the best candidates for inhibiting AuNPs aggregation propensity before and after freeze-drying. For this reason, **3**@AuNPs and **7**@AuNPs were selected for a further functionalization with biotinylated peptides **9** and **10** (Table 1), respectively. Also in this case, the ligand exchange method was employed. Three different ratios of non-biotinylated **3** and **7** peptides and biotinylated **9** and **10** ones were considered (80:20, 90:10, 95:5). All the analyzed ratios showed the ability to stabilize AuNPs in solution (see Supplementary Figure S2 in SI), so the 80:20 one, having the highest percentage of biotinylated peptide (20%), was selected for further evaluations.

**FIGURE 4:**
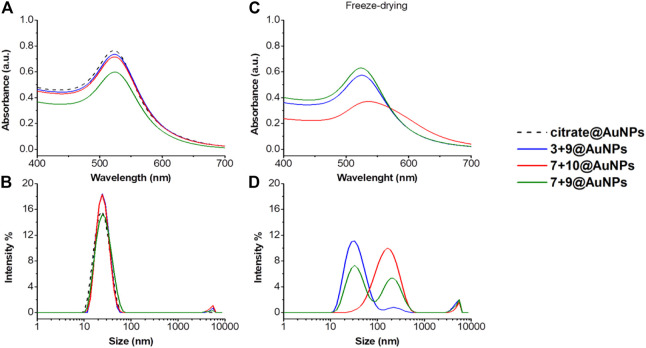
**(A)** Absorption spectra and **(B)** size distribution by intensity of citrate@AuNPs (dash) and functionalized-AuNPs with peptides **3 + 9** (blue), **7 + 10** (red), **7 + 9** (green) ratio 80:20. Mean DLS results are given for three different measurements. The hydrodynamic diameter is found 22.7 ± 0.2 nm PdI 0.2 ± 0.02 for citrate@AuNPs, 23.7 ± 0.6 nm PdI 0.2 ± 0.05 for **3 + 9**@AuNPs, 24.3 ± 0.6 nm PdI 0.2 ± 0.02 for **7 + 10**@AuNPs, and 23.4 ± 0.1 nm PdI 0.2 ± 0.02 for **7 + 9**@AuNPs. **(C)** Absorption spectra and **(D)** size distribution by intensity of functionalized-AuNPs after freeze-drying. Mean DLS results are given for three different measurements. The hydrodynamic diameter is found 33.9 ± 0.1 nm PdI 0.4 ± 0.03 for **3 + 9**@AuNPs, 140.3 ± 1.8 nm PdI 0.4 ± 0.02 for peptide **7 + 10**@AuNPs, and 37.6 ± 1.9 and 217.8 ± 20.6 nm PdI 0.6 ± 0.2 for peptide **7 + 9**@AuNPs.

**FIGURE 5:**
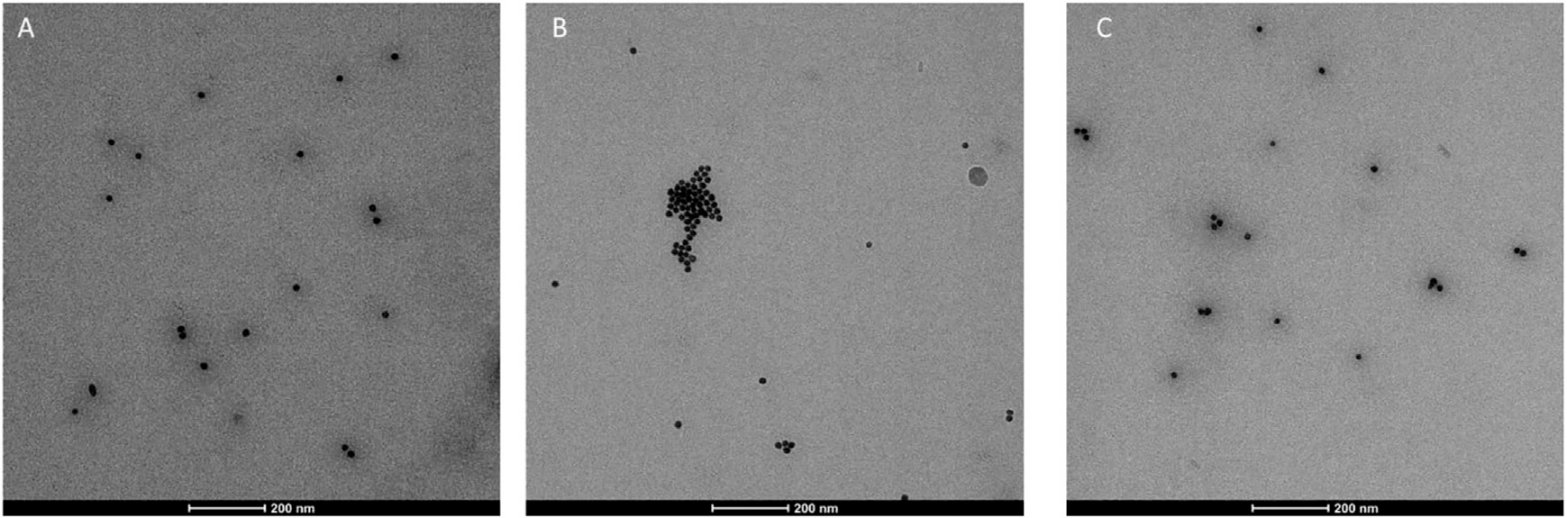
TEM micrographs of **(A) 3 + 9**@AuNPs, **(B) 7 + 10**@AuNPs, **(C) 7 + 9**@AuNPs.

Also in this case, the colloidal stability after freeze-drying of functionalized AuNPs was evaluated. As shown in Figure 5, only **3 + 9**@AuNPs show good colloidal stability while **7 + 10**@AuNPs aggregated. To overcome colloidal instability of **7 + 10**@AuNPs, Ac5c peptide **10** was replaced Aib peptide **9** (**7 + 9**@AuNPs). This substitution increased colloidal stabilization, as demostrated by the SPR peak that remained unchanged. This finding confirmed the higher efficacy of Aib containing peptides in inhibiting AuNPs aggregation than Ac5c ones. However, the DLS size distribution analysis showed, beyond the expected peak around 20–30 nm, a second peak of more than 200 nm, suggesting a partial aggregation of the particles. (Figure 4).

TEM analysis on biotinylated **3 + 9**@AuNPs, **7 + 10**@AuNPs, and **7 + 9**@AuNPs was then undertaken. As reported in Figure 5, a better NPs dispersion was observed for **3 + 9**@AuNPs (Figure 5A), while in the case of **7 + 10**@AuNPs the presence of aggregates was observed, confirming the UV-Vis and DLS data.

### Stability of Functionalized AuNPs in Physiological Environment

**FIGURE 6:**
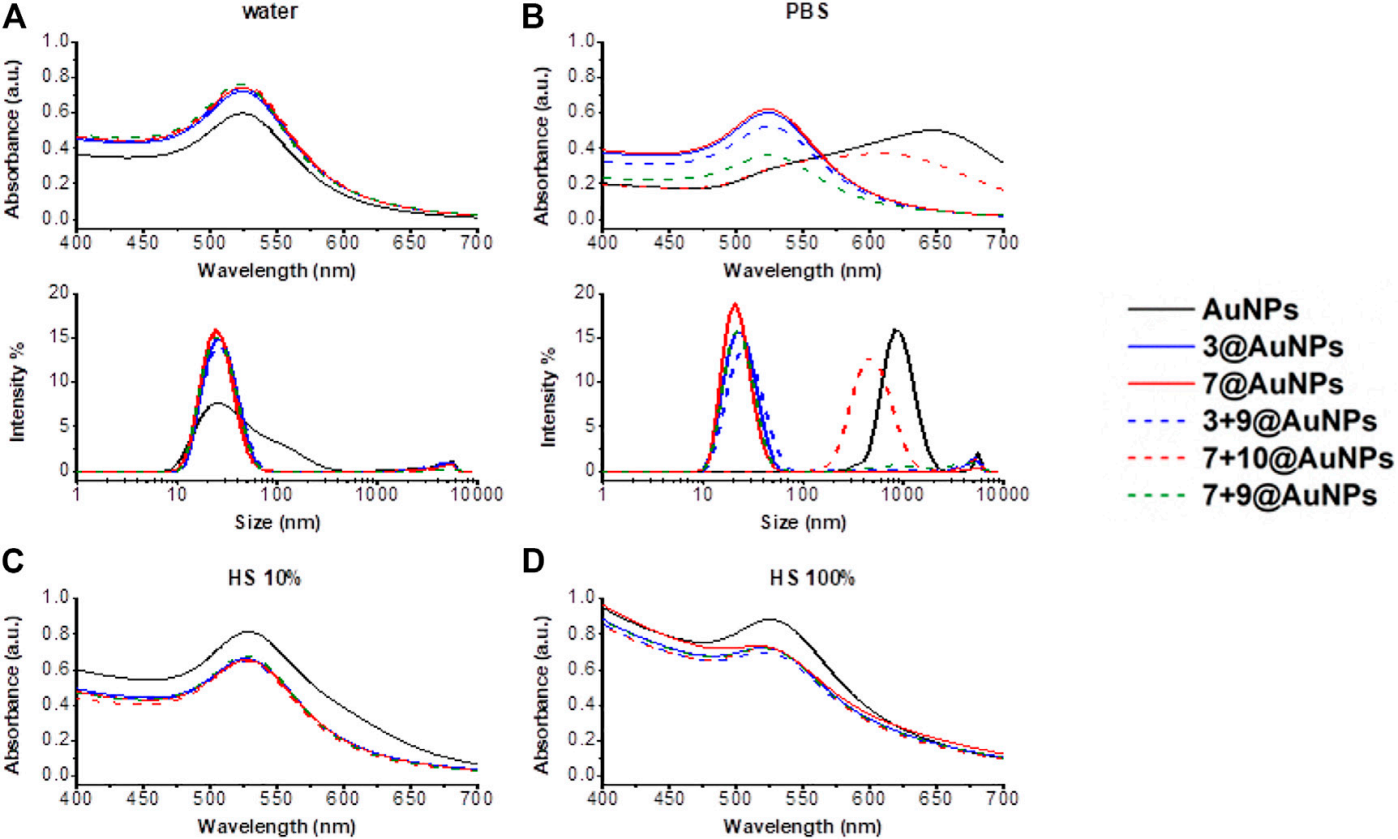
Stability study in different mediums. Absorption spectra and size distribution by intensity in **(A)** water, **(B)** PBS 1X and absorption spectra in **(C)** HS 10% in water and **(D)** HS 100% of AuNPs (black) and functionalized-AuNPs with peptides **3** (blue), **7** (red), **3 + 9** (dash blue), **7 + 10** (dash red), and **7 + 9** (dash green). Mean DLS results are given for three different measurements. A) The hydrodynamic diameter in water is found 31.0 ± 0.6 nm PdI 0.4 ± 0.01 for AuNPs, 25.5 ± 0.3 nm PdI 0.2 ± 0.002 for **3**@AuNPs, 23.7 ± 0.3 nm PdI 0.2 ± 0.03 for **7**@AuNPs, 26.3 ± 0.2 nm PdI 0.3 ± 0.02 for **3 + 9**@AuNPs, 23.8 ± 0.2 nm PdI 0.2 ± 0.02 for **7 + 10**@AuNPs, and 23.8 ± 0.4 nm PdI 0.2 ± 0.02 for **7 + 9**@AuNPs. B) The hydrodynamic diameter in PBS 1X is found 973.1 ± 14.2 nm PdI 0.4 ± 0.05 for AuNPs, 22.4 ± 0.6 nm PdI 0.3 ± 0.06 for **3**@AuNPs, 28.5 ± 0.6 nm PdI 0.2 ± 0.05 for **7**@AuNPs, 26.6 ± 0.6 nm PdI 0.3 ± 0.02 for **3 + 9**@AuNPs, 470 ± 35.9 nm PdI 0.2 ± 0.02 for **7 + 10**@AuNPs, and 23.5 ± 1.2 nm PdI 0.3 ± 0.03 for **7 + 9**@AuNPs.

The colloidal stability of AuNPs in a physiological environment is a fundamental prerequisite for biological applications. Three different mediums (water, PBS, 10% HS in water and 100% HS) were then considered and the peptide stability of functionalized-AuNPs was evaluated against naked AuNPs. Apart from the **7 + 10**@AuNPs in PBS, in all other cases, the functionalization with peptides ensured the dispersion stability of AuNPs and the DLS profile remained the same for weeks (see Supplementary Figure S3 in SI). As expected, in water, all functionalized-AuNPs showed a greater stability than naked AuNPs (Figure 6A). The major differences in dispersion stability were found in PBS.

PBS is a buffer solution frequently utilized as stimulated human body fluid in biological research. As already known from the literature (Du et al., 2012), when naked AuNPs were dispersed in PBS, the relatively high ionic strength could cause particle destabilization leading both to aggregation and adhesion to surrounding substrates through van der Waals forces. However, the functionalization of AuNPs with peptides **3** and **7** and with the mixture of **3 + 9** induced a great improvement of particle stability in PBS (Figure 6B), since no detectable changes in the UV-Vis absorbance and DLS spectra were observed. This is due to the local change of dielectric permittivity, resulting from the formation of a peptide layer on the surface of the particles. Thus, the peptide provides immediate protection to the particles from the aggregation. On the other hand, when the AuNPs were functionalized with the mixture of **7 + 10**, the destabilization occurred. Also in this case, the substitution of peptide **10** with Aib peptide **9** (mixture **7 + 9**) increased the particles stability in PBS, confirming the detrimental effect of Ac5c in the peptide sequence. In HS, the abundance of serum proteins hampered DLS analysis. Spectroscopic analyses, instead, showed interesting results. In fact, when uncapped AuNPs were dispersed in HS, both 10% in water and 100%, (Figures 6C,D) a shift of UV-Vis plasmon absorption of 10 nm (from 518 to 529 nm) was observed suggesting an increment of particle size due to the great amount of serum proteins conjugation to the particle surface. The shift was, instead, absent in all the peptide capped-AuNPs, that showed a better stability both in 10 and 100% HS. However, the broader SPR peak suggested the possibility of unspecific binding of proteins, also in presence of functionalized-AuNPs.

The “aging” of AuNPs over the time was recorded in all the considered mediums (see Supplementary Figure S3 in SI). The stability study over 2 months in water showed a gradual degradation of functionalized AuNPs since the SPR peak decreased gradually as well as the count rate measured by DLS analysis. However, AuNPs aggregation was prevented: DLS analyses showed at any time points the same peak at around 20 nm. The degradation rate was faster in biotinylated than in non-biotinylated AuNPs. Same results were obtained also in 10% aqueous HS solution. In PBS, instead, particle destabilization occurred after 15 days, when adhesion to surrounding substrates was observed.

**FIGURE 7:**
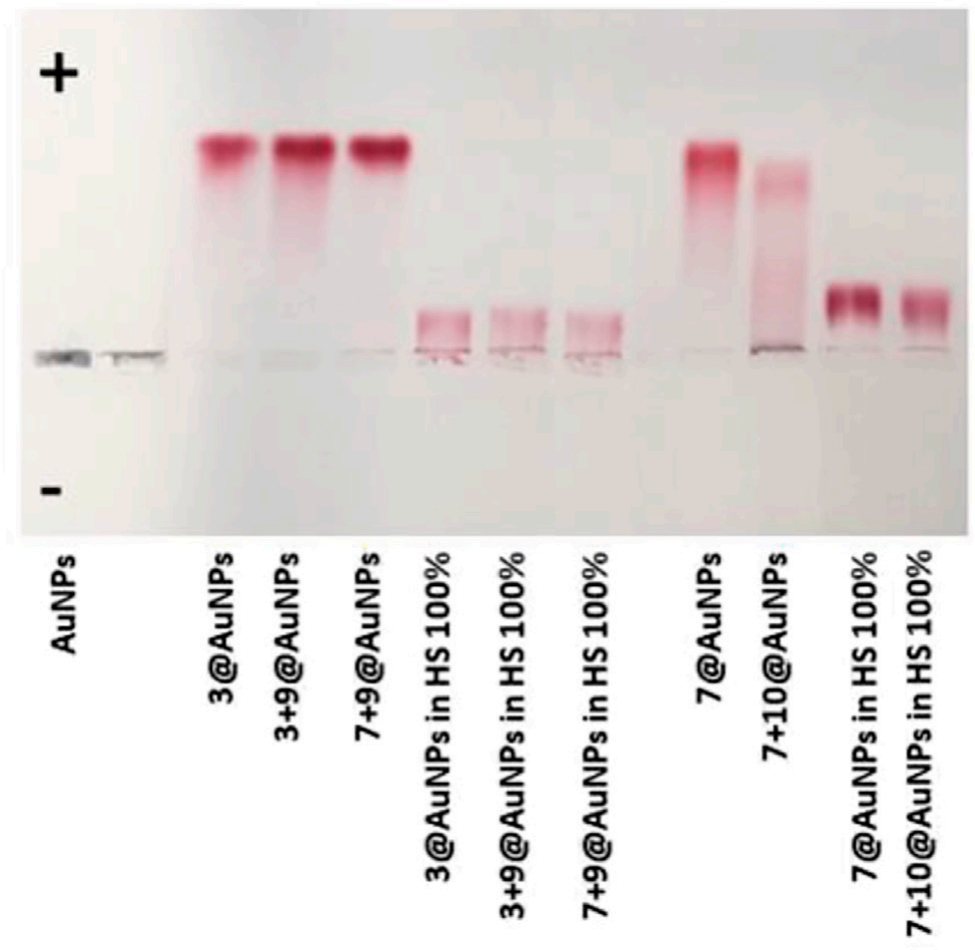
Agarose gel electrophoresis of AuNPs after incubation in water and in 100% HS.

Finally, agarose gel electrophoresis (Figure 7) was conducted on both peptide capped- and uncapped-AuNPs in order to confirm the previous data. All AuNPs were dispersed in water or incubated in HS 100% for 24 h at 37°C, followed by centrifugation in order to remove the incubation medium. Then, the particles were supplemented with 30% glycerol and loaded on agarose gel. Since the electrophoretic motilities are dependent on charge, size and isoelectric point of the particle, there was a distinct change in mobility of unstable and stable AuNPs. It is not surprising that AuNPs washed from the excess of citrate did not migrate over the gel, since they lost the negative charge of citrate, almost reaching a neutral charge. The dark color of AuNPs corroborated the formation of big aggregates and the loss of stability. All the peptide-functionalized AuNPs migrated to the cathode but, also in this case,**7 + 10**@AuNPs showed only a partial stability since a dark band was visible in the well of the gel and the red band was consequently less intensive. The substitution of **10** with **9** (**7 + 9**@AuNPs), once again, overcame the instability, confirming the data previous reported. Finally, when capped-AuNPs were dispersed in HS 100%, the gel migration decreased due to the high concentration of proteins that masked the particles’ charge. The capped-AuNPs appeared stable but, probably, a partial unspecific binding of proteins occurred.

### Avidin Specific Recognition

First, the interaction of biotinylated peptides with avidin was assessed by the HABA (4′-hydroxyazobenzene-2-carboxylic acid) test that allowed also to evaluate the number of peptides per particle (see Supplementary Figure S2 and text in SI for details). HABA is a dye that enables spectrophotometric estimation of biotinylation levels of labeled molecules. A total of peptides was calculated to be 4.0 × 10^15^ biotinylated molecules for **3 + 9**@AuNPs and 3.6 × 10^15^ biotinylated molecules for **7 + 10**@AuNPs. This translates to 370 molecules of peptide **9** per nanoparticle and to 334 molecules of peptide **10** per nanoparticle.

**FIGURE 8:**
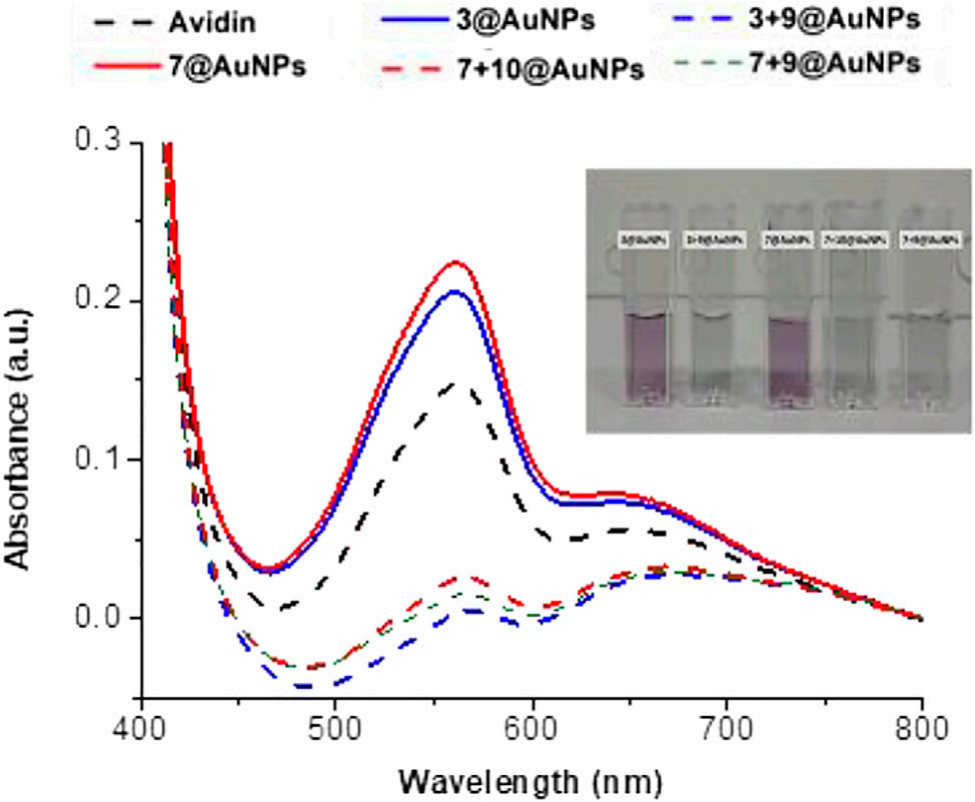
Evaluation of avidin interaction with non-biotinylated and biotinylated AuNPs analyzing washing supernatant by BCA assay.

The specific binding of avidin to biotinylated AuNPs was further evaluated with BCA (Bicinchoninic Acid) Protein assay. The principle of this method is the biuret reaction by which proteins can reduce Cu^+2^ to Cu^+1^ in an alkaline solution and results in a purple color formation by bicinchoninic acid. Avidin aqueous solution (0.125 mg/ml) was added to biotinylated AuNPs (**3 + 9**@AuNPs, **7 + 10**@AuNPs or **7 + 9**@AuNPs) and to non-biotinylated **3**@AuNPs or **7**@AuNPs. The mixtures were shacked for 2 h. Then, the mixture was centrifuged and the supernatant was analyzed calorimetrically by BCA assay. As shown in Figure 8, the presence of avidin in blank supernatant was evident both optically (the supernatant solutions analyzed with BCA assay acquired the typical purple color) and spectrophotometrically, while the supernatants of biotinylated AuNPs were completely free from avidin. These results suggested that nonspecific interaction of avidin with peptides capped-AuNPs was prevented and the avidin specific interaction occurred only in presence of biotin.

**FIGURE 9:**
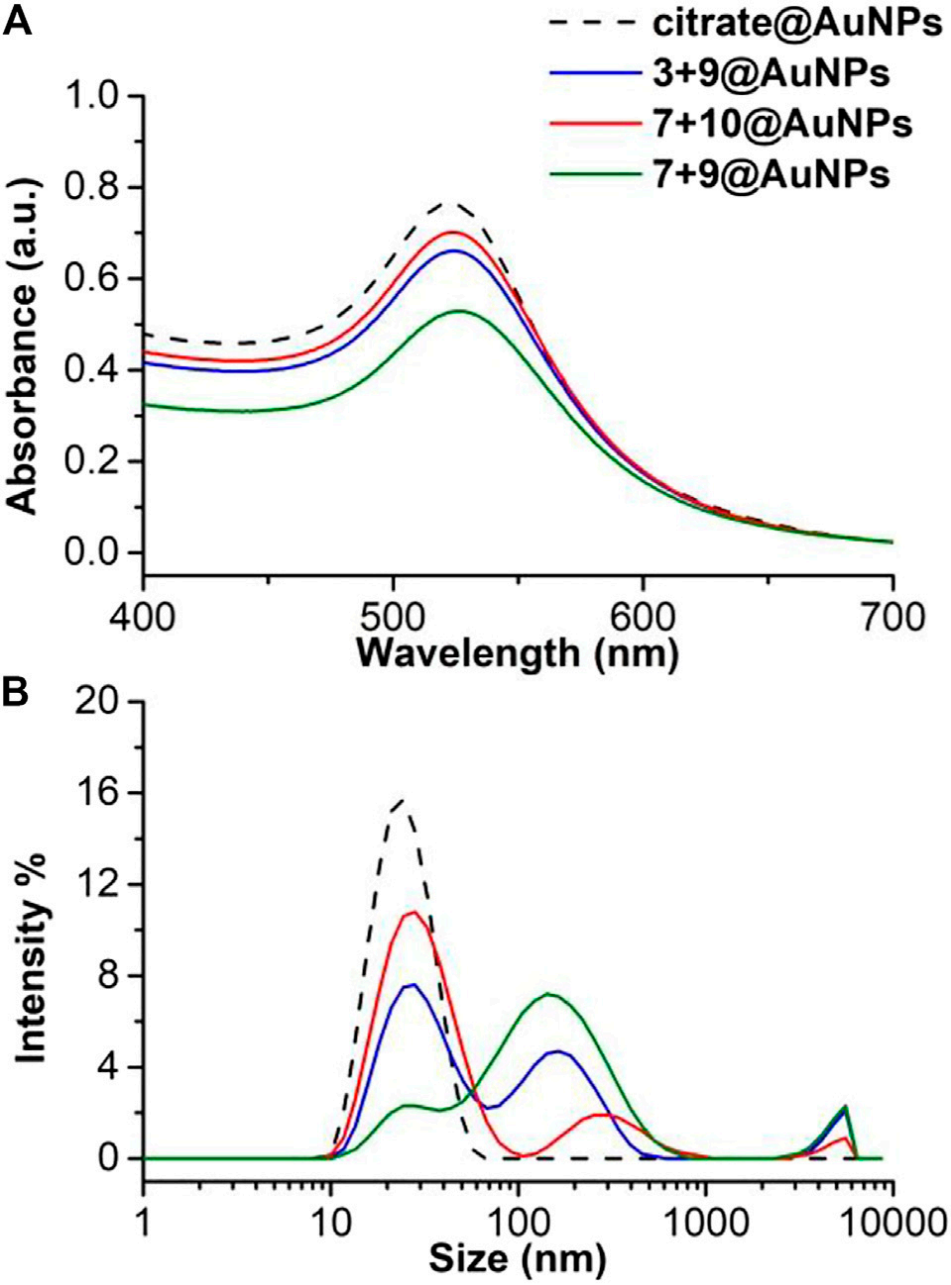
**(A)** Absorption spectra and **(B)** size distribution by intensity of citrate@AuNPs (dash) and functionalized-AuNPs with peptides **3 + 9** (blue), **7 + 10** (red), and **7 + 9** (green) after addition of avidin solution (0.0125 mg/ml). Mean DLS results are given for three different measurements. The hydrodynamic diameter is found 22.7 ± 0.2 nm PdI 0.2 ± 0.02 for citrate@AuNPs, 33 ± 4 and 176.7 ± 19.8 nm PdI 0.6 ± 0.1 for **3 + 9**@AuNPs, 31.4 ± 1.9 and 294.1 ± 3.4 nm PdI 0.4 ± 0.03 for **7 + 10**@AuNPs, and 25.9 ± 0.5 and 158.2 ± 8 nm PdI 0.6 ± 0.09 for **7 + 9**@AuNPs.

The addition of avidin to the biotinylated AuNPs resulted in particle aggregation and the redispersion in water was no possible, thus confirming the accessibility and recognition of the biotin moieties. This aggregation happened because avidin can bind up four biotin molecules since it has two pairs of biotin binding sites on opposite sides of the molecule (Aslan et al., 2004). The aggregate grows only when different biotinylated AuNPs cross-linked with the same avidin molecule. Therefore, the concentration of avidin solution was decreased up to 0.0125 mg/ml to reduce the possibility of cross-linking between the particles. The avidin-biotinylated AuNPs interaction was analyzed by UV-Vis and DLS (Figure 9). The UV-Vis measurements (Figure 9A) gave a single sharp SPR peak signifying that the integrity of the biotinylated AuNPs was maintained. On the other hand, DLS analysis (Figure 9B) showed two peaks: one at ∼ 20 nm corresponding to the peak of AuNPs, the other at ∼ 200 nm suggesting a partial aggregation probably induced by biotin-avidin interaction. The aggregation is more evident with **7 + 9**@AuNPs and **3 + 9**@AuNPs, both with the biotinylated Aib peptide **9**.

**FIGURE 10:**
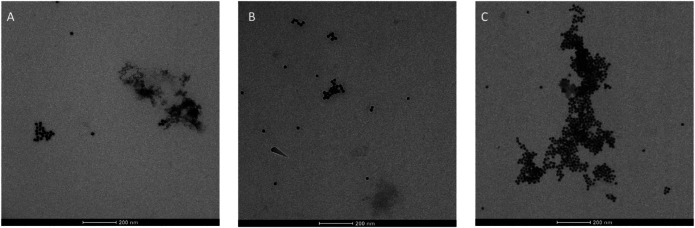
TEM micrographs of avidin bound: **(A) 3 + 9**@AuNPs, **(B) 7 + 10**@AuNPs, **(C) 7 + 9**@AuNPs.

Finally, TEM analysis was performed on avidin bound biotinylated **3 + 9**@AuNPs, **7 + 10**@AuNPs, and **7 + 9**@AuNPs confirming the formation of large NPs aggregates (Figure 10) and thus avidin binding. As previously observed in DLS experiments, the aggregates were particularly relevant in the case of **7 + 9**@AuNPs.

## Conclusion

In this work, a small library of ultrashort peptides containing alanine and two different constrained AAs (Aib or Ac5c) was evaluated as inhibitors of AuNPs aggregation. Apart from AA composition, the anchor point (at C- or N-terminus) and the peptide length were also considered through UV-Vis, DLS and TEM experiments. Peptide **3,** containing 2 Aib residues and the *N*-terminus thiopropionate capping moiety, was demonstrated as the most effective tool for the colloidal stabilization of AuNPs, also in physiological environment. Of relevance, **3**@AuNPs were found storable as lyophilized powders without losing the stabilization properties once re-dispersed in water. On the other hand, Ac5c peptides were found less effective, suggesting that the higher steric hindrance of Ac5c with respect to Aib could have negative influence on the AuNPs stability.

Finally, the possibility to bind proteins on peptide capped AuNPs was evaluated using the biotin/avidin interaction as the model. **3**@AuNPs further functionalized with biotinylated peptide **9** allowed protein binding through molecular recognition, paving the way to the exploitation of these systems in nanomedical devices.

## Data Availability

The original contribution presented in the study are included in the article/Supplementary Material, further inquiries can be directed to the corresponding author.
