# Supplementary material for: Ultrashort Peptides and Gold Nanoparticles: Influence of Constrained Amino Acids on Colloidal Stability
Source: Front Chem. 2021 Oct 1;9:736519. doi: 10.3389/fchem.2021.736519 (PMC8517408; doi:10.3389/fchem.2021.736519)
Supplement: Supplementary file 1 [file Table1.DOCX]

Supplementary Material

General Synthetic Procedures 2

Secondary structure evaluation by FT-IR 5

# Functionalization of AuNPs with biotinylated peptides 6

# Stability studies 7

# Biotinylated peptide loading 10

# Copies of ^1^H-NMR, ^13^C-NMR spectra 12

#

# General Synthetic Procedures: Solid Phase Peptide Synthesis (SPPS)

**1.1 SPPS of Peptide 1- 10**

The peptides were synthesised on Rink Amide MHBA resin (100-200 mesh, loading 0.69 mmol/g), 0.15 mmol scale, using the manual solid-phase peptide synthesis (SPPS) Fmoc/t-Bu strategy.

0,217 g of the resin was swollen in a mixture of DCM (6.5 mL) and NMP (1 mL) for 30 min at room temperature (RT) before use. Each Fmoc group removal was performed by treatment of the resin with a solution of 20% piperidine (v/v) in DMF (5+15 min). Then, then resin was washed 6 times with DMF.

The coupling reactions for the first two amino acids and for the third ones of peptides 1, 4, 6, 9 and 10were performed in DMF (1.1 ml) using as coupling system 3:5:5:8 equivalents of Fmoc-amino acid/HOBt/HBTU/DIEA. The mixture was shaked for 1 hour at RT and after that, the resin was washed 6 times with DMF. The incorporation of each amino acid was confirmed by the Kaiser test, indicating for all the couplings a complete reaction.

The other couplings were performed in DMF (1.1 ml) using as coupling system 3:5:5 equivalents of Fmoc-amino acid/HOBt/DIC. The mixture was shaked for 1 hour at RT and after that, washed 6 times with DMF and 6 times with DCM. The incorporation of each amino acid was confirmed by the Kaiser test, indicating for all the couplings a complete reaction.

The N-terminus of peptide 1,2,4,6 was acetylated. The capping was performed in a mixture of DMC (1mL) and DMF (1 mL) using 0.1 ml of acetic anhydride and 0.2 ml of DIEA. The mixture was shacked for 30 minutes at RT, then, the resin was filtered, and the reaction was repeated for 30 min.

The N-terminus conjugation of peptide 3 and 7 to biotin, to give respectively peptide 9 and 10, was performed in DMF (1mL/100 mg of resin) using as coupling system 10:10:10 equivalents of biotin/DIC/Oxyma Pure. The coupling was carried out o.n., at R.T. and under gently shaking. The incorporation of biotin was monitored by Kaiser Test, indicating for both the couplings a complete reaction.

Finally, the resin was washed 6 times with DMF, 6 times with DCM and 6 times with diethyl ether.

**1.2 Cleavage of Peptide 1-10** The peptidyl bound resin was treated with a mixture (2 ml) of TFA/ phenol/ H_2_O/ thioanisole/ TIPS (84:5:5:5:1 v/v/v/v) for 2 hours under magnetic stirring. Then, the resin was filtered and washed with methanol. The filtrate was concentrated in vacuum and extruded in 40 ml of Et_2_O. The precipitate was collected by centrifugation (6000 rpm, 5 min). Then, the supernatant was decanted, the residue was resuspended in 20 ml of Et_2_O and centrifuged again. This procedure was repeated twice.

**1.3 Purification of Peptide 1-10**

The raw products were purified by semi-preparative RP-HPLC, using a DENALI C18 column (10µm, 250 x 22 mm). The solvent system used was: 95:5:0.1 H_2_O/CH_3_CN/TFA (v/v/v) (A) and 95:5:0.1 CH_3_CN/H_2_O/TFA (v/v/v) (B), the flow rate 20 mL/min, gradient from 0% to 50% of B in 20 min, λ 220 nm. After lyophilization, the peptides were characterized by ESI-MS mm and characterized by ^1^H-NMR and ^13^C-NMR.

**Table S1.**

|  |  | **MS-ESI (m/z)** | |
| --- | --- | --- | --- |
| **Compound** | **Sequence** | **Calculated** | **Found** |
| **Peptide 1** | Ac-A-Aib-A-Aib-A-C-NH_2_ | 545.26 | 545.9 [M+H]^+^ |
| **Peptide 2** | Ac-C-A-Aib-A-Aib-A-NH_2_ | 545.26 | 544.27 [M-H]^-^ |
| **Peptide 3** | HS-CH_2_CH_2_CO -A-Aib-A-Aib-A-NH_2_ | 488.24 [M+H]^+^ | 489.07 [M+H]^+^ |
| **Peptide 4** | Ac-A-Aib-A-C-NH_2_ | 389.17 | 413.25 [M+Na]^+^ |
| **Peptide 5** | HS-CH_2_CH_2_CO -A-Aib-A-NH_2_ | 332.15 [M+H]^+^ | 334.96 [M+H]^+^ |
| **Peptide 6** | Ac-A-Ac_5_c-A-Aib-A-C-NH_2_ | 571.28 | 594.2 [M+Na]^+^ |
| **Peptide 7** | HS-CH_2_CH_2_CO-A-Ac_5_c-A-Aib-A-NH_2_ | 514.26 [M+H]^+^ | 515.17 [M+H]^+^ |
| **Peptide 8** | HS-CH_2_CH_2_CO-A-Ac_5_c-A-NH_2_ | 358.17 | 357.12 [M-H]^-^ |
| **Peptide 9** | Biotin-A-Aib-A-Aib-A-C-NH_2_ | 729.33 [M+H]^+^ | 730.28 [M+H]^+^ |
| **Peptide 10** | Biotin-A-Ac_5_c-A-Aib-A-C-NH_2_ | 755.35 [M+H]^+^ | 756.36 [M+H]^+^ |

**Peptide 1**: ^1^H NMR (300 MHz, CD_3_OD) δ 1.36 (d, 3H, J=7.2 Hz), 1.41 (d, 3H, J=7.3 Hz), 1.43 (d, 6H, J=11.2 Hz), 1.47-1.50 (m, 9H), 2.04 (s, 3H), 2.98-3.01 (m, 2H), 4.00-4.22 (m, 3H), 4.31-4.36 (m, 1H). ^13^C NMR (75 MHz, CD_3_OD) δ 15.29, 15.51, 15.81, 21.04, 22.54, 22.89, 24.87, 25.17, 25.34, 50.80, 50.93, 51.88, 56.07, 56.42, 56.70, 172.65, 173.96, 174.12, 174.54, 175.06, 176.55, 176.79.

**Peptide 2**: ^1^H NMR (300 MHz, CD_3_OD) δ 1.39-1.46 (m, 15H), 1.49 (s, 6H), 2.01 (s, 3H), 2.85-2.87 (m, 2H), 3.97-4.06 (m, 1H), 4.10-4.17 (m, 1H), 4.19-4.27 (m, 1H), 4.43-4.47 (m, 1H). ^13^C NMR (75 MHz, CD_3_OD) δ 15.33, 15.45, 16.21, 21.04, 22.80, 23.08, 24.96, 25.06, 25.2, 49.49, 51.17, 51.42, 55.77, 56.26, 56.58, 171.77, 172.16, 173.82, 174.51, 175.59, 176.25, 176.94.

**Peptide 3**: ^1^H NMR (300 MHz, CD_3_OD) δ 1.38 (d, 3H, J= 7.3 Hz), 1.41-1.45 (m, 9H), 1.49 (d, 3H, J=1.1 Hz), 2.55-2.61 (m, 2H), 2.77-2.81 (m, 2H), 4.02-4.13 (m, 2H), 4.18-4.25 (m, 1H). ^13^C NMR (75 MHz, CD_3_OD) δ 15.33, 15.41, 16.16, 19.56, 22.81, 23.01, 25.09, 25.32, 38.65, 49.53, 51.31, 51.47, 56.19, 56.59, 173.41, 174.21, 174.65, 175.65, 176.36, 177.00.

**Peptide 4**: ^1^H NMR (300 MHz, CD_3_OD) δ 1.35 (d, 3H, J=1.35 Hz), 1.44 (s, 6H), 1.51 (d, 3H, J=1.51 Hz), 2.85-3.11 (m, 2H), 4.10-4.20 (m, 2H), 4.35-4.40 (m, 1H), 7.81, 7.96, 8.36. ^13^C NMR (75 MHz, CD_3_OD) δ 15.58, 15.71, 20.98, 22.58, 23.24, 25.26, 50.12, 51.00, 56.28, 56.39, 172.29, 172.39, 174.17, 174.70, 176.83.

**Peptide 5**: ^1^H NMR (300 MHz, CD_3_OD) δ 1.37 (d, 3H, J=7.2 Hz), 1.42 (d, 3H, J=7.3 Hz), 1.47 (d, 6H, J=7.4 Hz), 2.56 (m, 2H), 2.78 (m, 2H), 4.16-4.20 (m, 1H), 4.23-4.29 (m, 1H), 7.75, 8.11, 8.35. ^13^C NMR (75 MHz, CD_3_OD) δ 15.54, 16.25, 19.53, 23.62, 24.53, 38.89, 49.42, 50.53, 56.44, 173.00, 174.13, 175.20, 176.76.

**Peptide 6**: ^1^H NMR (300 MHz, CD_3_OD) δ 1.36 (d, 3H, J=7.3 Hz), 1.39 (d, 3H, J= 7.4 Hz), 1.47 (s, 3H), 1.48 (d, 3H, J= 7.4 Hz), 1.51 (s, 3H), 1.72-1.83 (m, 5H), 1.96-1.99 (m, 2H), 2.01 (s, 3H), 2.41-2.50 (m, 1H), 2.98-3.00 (m, 2H), 4.04-4.12 (m, 2H), 4.17-4.20 (m, 1H), 4.32-4.37 (m, 1H), 7.89, 8.27. ^13^C NMR (75 MHz, CD_3_OD) δ 15.26, 15.43, 15.84, 21.03, 22.53, 23.94, 24.02, 25.19, 25.33, 35.92, 36.89, 50.81, 50.90, 51.95, 56.43, 56.68, 66.33, 172.73, 173.93, 174.51, 174.62, 175.04, 176.11, 176.70.

**Peptide 7**: ^1^H NMR (300 MHz, CD_3_OD) δ 1.37 (d, 3H, J= 7.2 Hz), 1.41 (d, 3H, J= 7.3 Hz), 1.43 (d, 3, J=7.3 Hz), 1.48 (s, 3H), 1.49 (s, 3H), 1.72-1.77 (m, 4H), 1.79-1.89 (m, 1H), 1.94-1.98 (m, 2H) 2.40-2.47 (m, 1H), 2.55-2.61 (m, 2H), 2.76-2.82 (m, 2H), 4.01-4.15 (m, 2H), 4.18-4.25 (m, 1H). ^13^C NMR (75 MHz, CD_3_OD) δ 15.30, 15.40, 16.16, 19.63, 23.06, 24.07, 24.21, 25.30, 36.13, 37.21, 38.69, 49.57, 51.17, 51.48, 56.57, 66.34, 172.41, 173.45, 174.59, 175.59, 175.93, 176.96.

**Peptide 8**: ^1^H NMR (300 MHz, CD_3_OD) δ 1.37 (d, 3H, J=7.2 Hz), 1.42 (d, 3H, J=7.2 Hz), 1.76-1.79 (m, 4H), 1.90-2.02 (m, 2H), 2.06-2.13 (m, 1H), 2.30-2.34 (m, 1H), 2.55-4.59 (m, 2H), 2.75-2.80 (m, 1H), 4.13-4.21 (m, 1H), 4.25-4.30 (m, 1H). ^13^C NMR (75 MHz, CD_3_OD) δ 15.47, 16.34, 19.58, 24.12, 24.13, 36.58, 36.79, 38.87, 49.59, 50.46, 66.62, 173.06, 174.59, 174.81, 176.78.

**Peptide 9**: ^1^H NMR (400 MHz, CD_3_OD) δ 8.24 (s, 1H), 8.10 – 8.06 (m, 1H), 7.98 (s, 1H), 7.93 – 7.82 (m, 1H), 4.56 – 4.44 (m, 1H), 4.41 – 4.26 (m, 2H), 4.24 – 4.15 (m, 1H), 4.15 – 4.00 (m, 2H), 3.25 – 3.15 (m, 1H), 3.04 – 2.86 (m, 3H), 2.71 (d, 1H), 2.36 – 2.24 (m, 2H), 1.85 – 1.54 (m, 5H), 1.54 – 1.33 (m, 23H). ^13^C NMR (100 MHz, CD_3_OD) δ 176.81, 176.61, 175.32, 175.16, 174.58, 174.28, 174.00, 164.67, 62.07, 60.24, 56.73, 56.44, 56.10, 55.67, 51.97, 51.00, 50.83, 39.62, 34.61, 28.47, 28.24, 25.43, 25.20, 25.17, 24.96, 22.98, 22.55, 16.95, 15.87, 15.43.

**Peptide 10**: ^1^H NMR (400 MHz, CD_3_OD) δ 4.60 – 4.46 (m, 1H), 4.45 – 4.27 (m, 2H), 4.25 – 4.18 (m, 1H), 4.17 – 4.04 (m, 2H), 3.27 – 3.15 (m, 1H), 3.05 – 2.90 (m, 3H), 2.73 (d, *J* = 12.7 Hz, 1H), 2.52 – 2.41 (m, 1H), 2.40 – 2.26 (m, 2H), 2.14 – 1.92 (m, 2H), 1.93 – 1.83 (m, 1H), 1.83 – 1.57 (m, 8H), 1.54 – 1.33 (m, 17H).^13^C NMR (100 MHz, CD_3_OD) δ 176.78, 176.21, 175.38, 175.20, 174.76, 174.60, 66.31, 62.06, 60.25, 56.76, 56.41, 55.67, 52.08, 50.91, 50.85, 50.64, 49.37, 39.62, 36.98, 36.14, 34.58, 28.44, 28.23, 25.44, 25.22, 25.17, 24.15, 24.04, 22.54, 15.90, 15.42, 15.34.

# Secondary structure evaluation

FTIR analyses were performed on compounds **3**, **9**, **7** and **10**. As we expected, all studied peptides possess only helical conformation [A. Barth,Biochim. Biophys. Acta, Bioenerg., 2007,1767, 1073–1101].as already reported for similar compounds [ref].


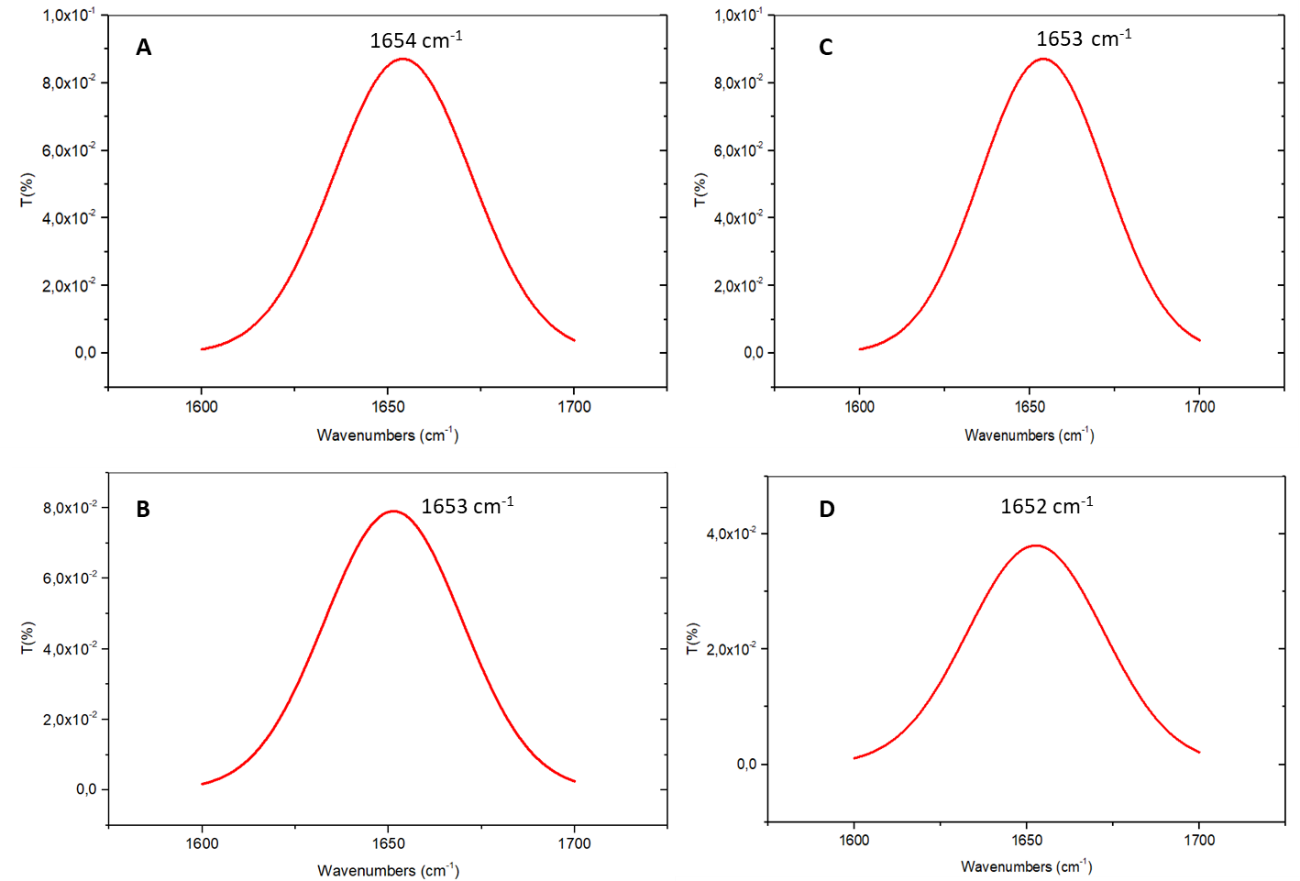


**Figure S1** Amide I Stretching band region of compounds **3** (A), **7** (B), **9** (C), **10** (D)

# Functionalization of AuNPs with biotinylated peptides


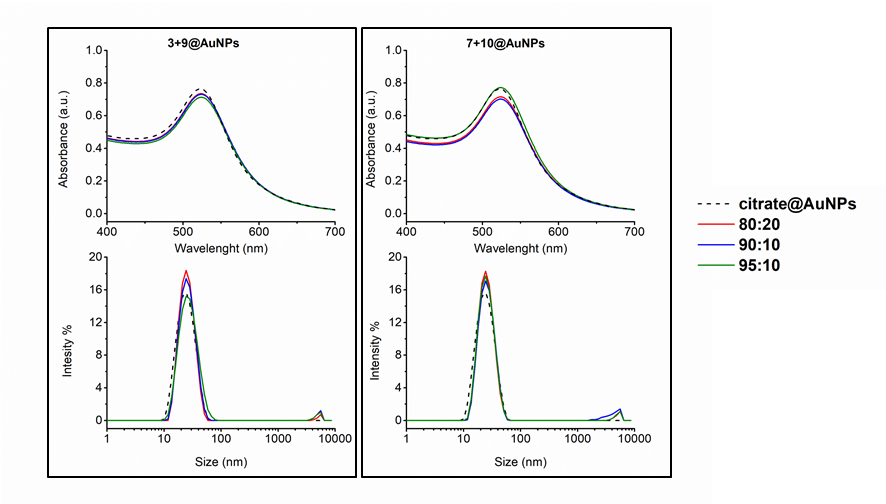


**Figure S2.** Size distribution by intensity and absorption spectra of citrate@AuNPs (dash) and functionalized-AuNPs with different ratios of peptide mixtures **3+9** and **7+10**: 80:20 (red), 90:10 (blue) and 95:5 (green). Mean DLS results are given for three different measurements. The hydrodynamic diameter is found 22.7±0.2 nm PdI 0.2±0.02 for citrate@AuNPs, peptide **3+9**@AuNPs: 23.7±0.6 nm PdI 0.2±0.05 for 80:20, 24.6±0.6 nm PdI 0.3±0.04 for 90:10, 24.8±0.4 nm PdI 0.3±0.03 for 95:5. Peptide **7+10**@AuNPs: 24.3±0.6 nm PdI 0.2±0.02 for 80:20, 25.1±0.3 nm PdI 0.3±0.02 for 90:10, 24.0±0.5 nm PdI 0.2±0.04 for 95:5.

# Stability studies


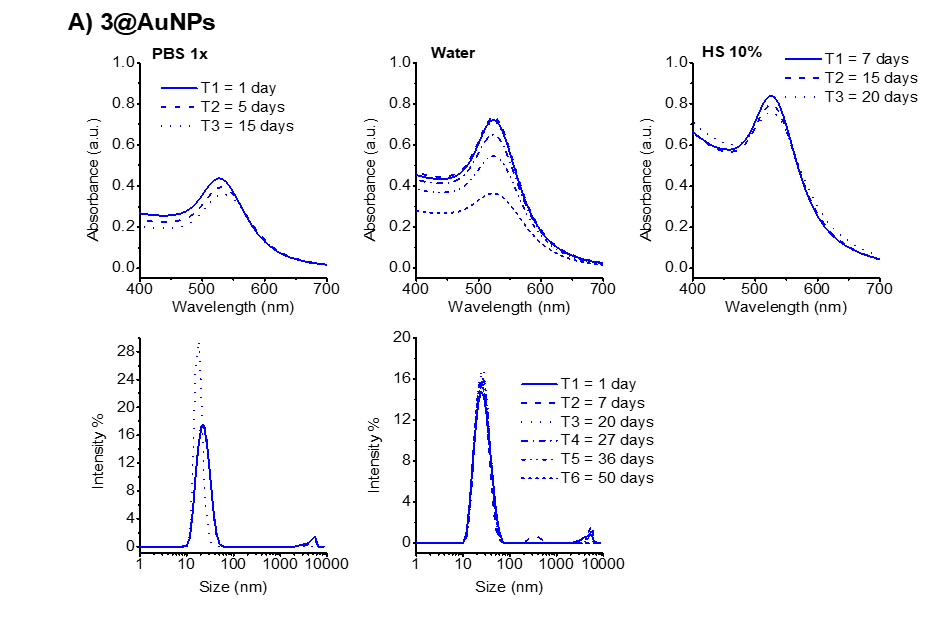

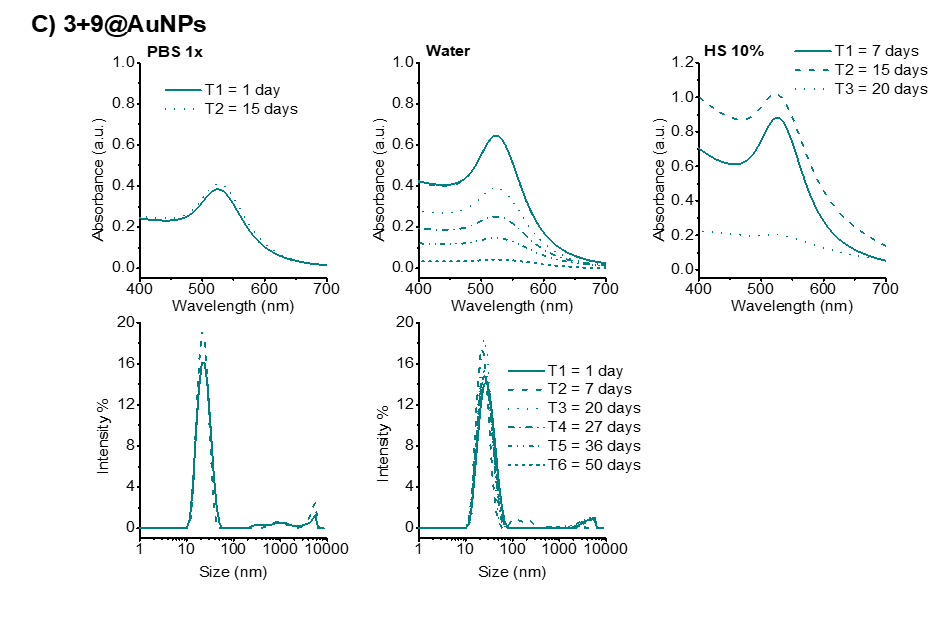

**Figures S3.** Stability studies over the time of A) **3**@AuNPs, B) **7**@AuNPs, C) **3+9**@AuNPs, D) **7+10**@AuNPs, E) **7+9**@AuNPs in PBS 1X, water and HS 10%.

# Biotinylated peptide loading

HABA dye (4'-hydroxyazobenzene-2-carboxylic acid) binds to avidin to produce a yellow-orange colored complex which absorbs at 500 nm. Free biotin will displace the HABA dye and cause the absorbance to decrease. A negative and a positive control can be prepared to estimate the amount of biotin, and subsequently, the number of biotinylated peptides per AuNPs. For the negative control, 500 μl of avidin 0.5 mg/ml in water were added to 15 μl of HABA solution (2.42 mg/ml in 10 mM NaOH), then, the mixture was shacked for 5 minutes at room temperature and the absorption spectrum was acquired. For the positive control, peptide **9** or **10** 0.5 mM solution in DMSO were diluted with water in order to reach a concentration of 8.75 μM. Then, 10 μl of peptide **9** or **10** 8.75 μM solution were added to 90 μl of HABA-avidin solution. The mixture was shacked for 5 minutes and then, the absorption spectrum was acquired.

To determine the amount of biotinylated peptide per particle, **3+9**@AuNPs and **7+10**@AuNPs (2ml, 0.9 nM in water) were prepared according to the method described in paragraph 2.3 of the main text. After washing by centrifugation, 10 μl of **3+9**@AuNPs and **7+10**@AuNPs (0.9 nM) were added to 90 μl of HABA-avidin solution. The mixture was shacked for 5 minutes and then, the absorption spectrum was acquired. The data are shown in figure SI2. Both **3+9**@AuNPs and **3+10**@AuNPs were able to displace HABA and bind avidin. In fact, the absorbance at 500 nm of HABA-avidin complex decreased, respect to the negative control, in presence of functionalized AuNPs, while the peak at 350 nm of the free HABA increased. As expected, since not all peptides in the functionalized mixture were able to bind the AuNPs, the absorbance at 500 nm of positive control was even lower than the absorbance of functionalized AuNPs.

**Figure S4.** Absorption spectra of HABA-avidin complex acquired in absence of biotin molecules (negative control), and in presence of biotinylated peptides (positive control) and biotinylated AuNPs (**3+9**@AuNPs and **3+10**@AuNPs).

The total amount of biotinylated peptide molecules bonded to AuNPs (N_bbp_) were calculated according to the following equation:

**N_bbp_ = N_btot_ – (A_(bs)_/A_(neg ctr)_ · N_tot_)**

where N_btot_ is the total amount of biotinylated peptide molecules used in all experiments (1.05 x 10^16^), while A_(bs)_/A_(neg ctr)_ is the ratio between the absorbance at 500 nm of biotinylated AuNPs and negative control.

# Copies of ^1^H-NMR, ^13^C-NMR spectra

**Peptide 1**

Figure S5. Chemical structure of peptide 1 (Ac-A-Aib-A-Aib-A-C-NH_2_).


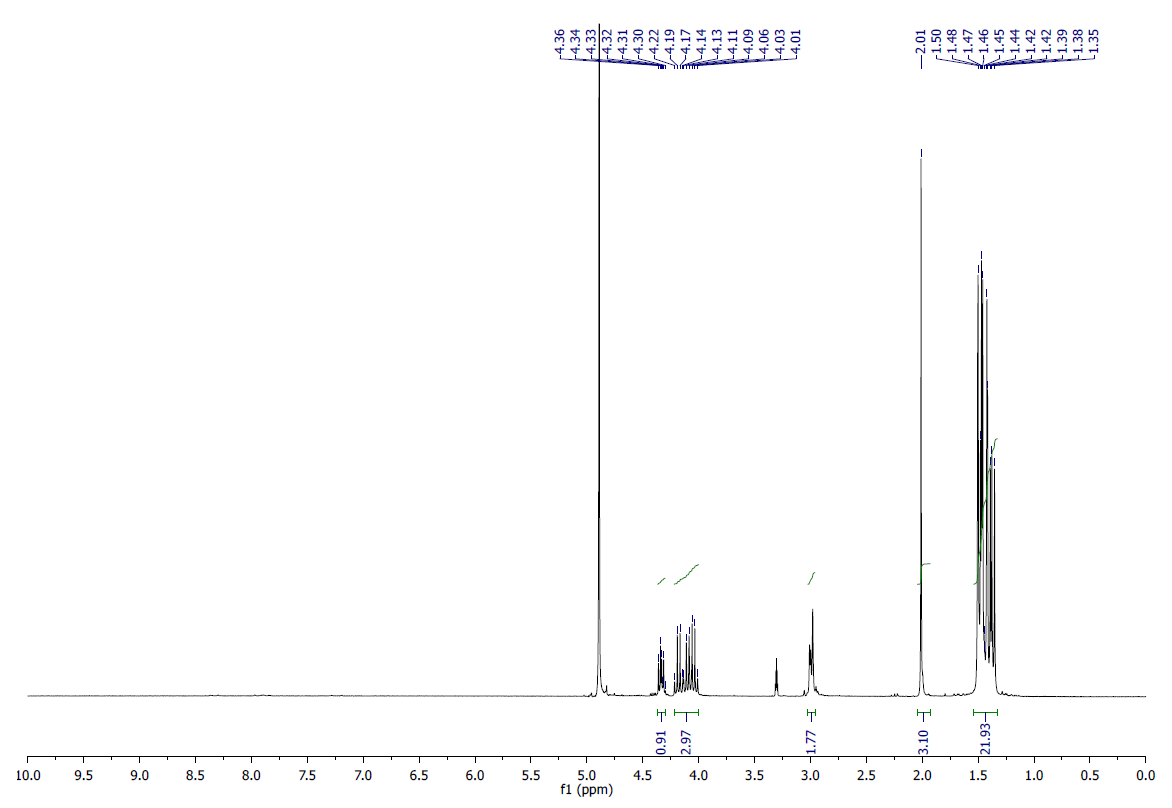
Figure S6. ^1^H-NMR of peptide 1 (300 MHz, CD_3_OD).


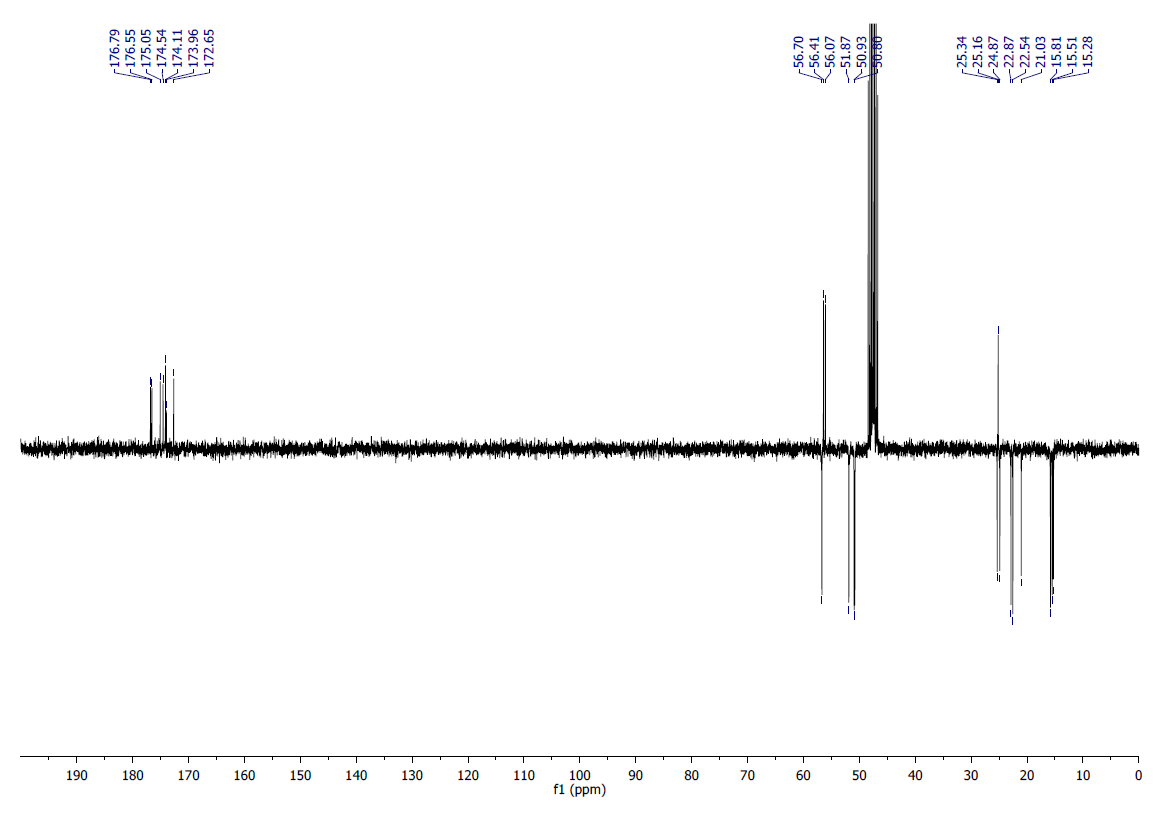
Figure S7. ^13^C-NMR of peptide 1 (75 MHz, CD_3_OD).

**Peptide 2**

Figure S8. Chemical structure of peptide 2 (Ac-C-A-Aib-A-Aib-A-NH_2_).


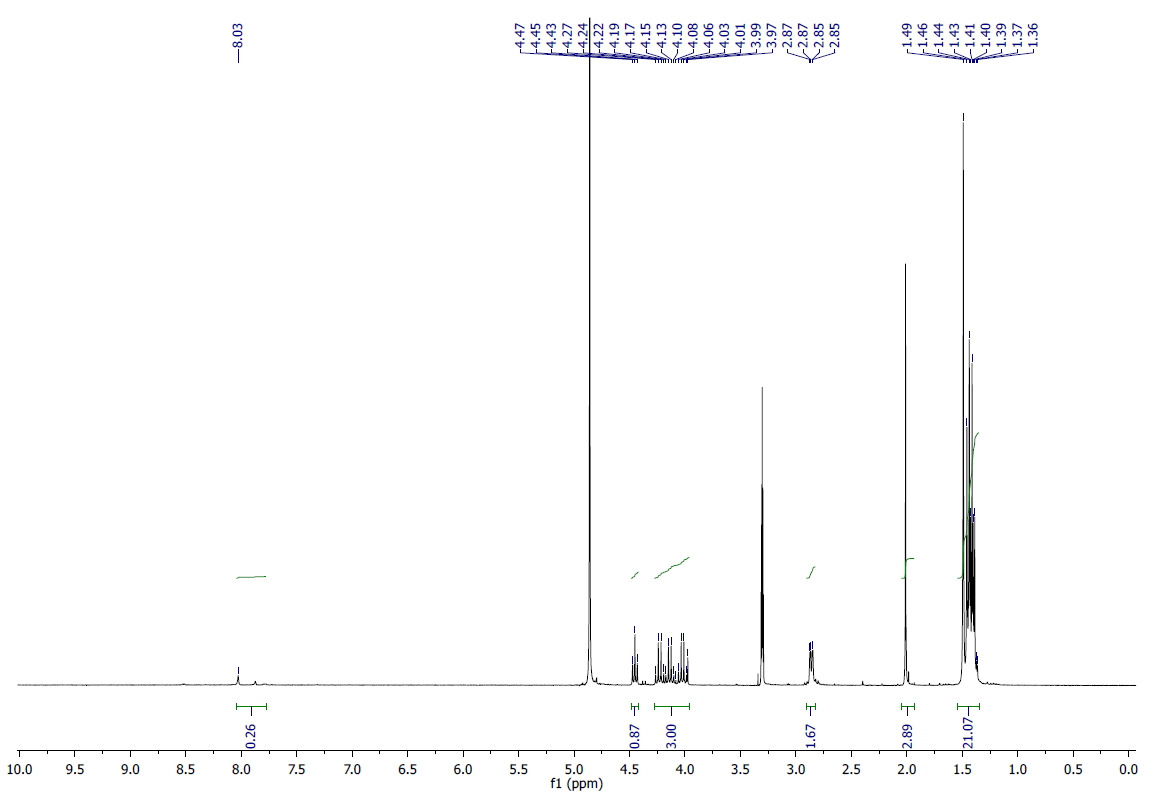
Figure S9. ^1^H-NMR of peptide 2 (300 MHz, CD_3_OD).


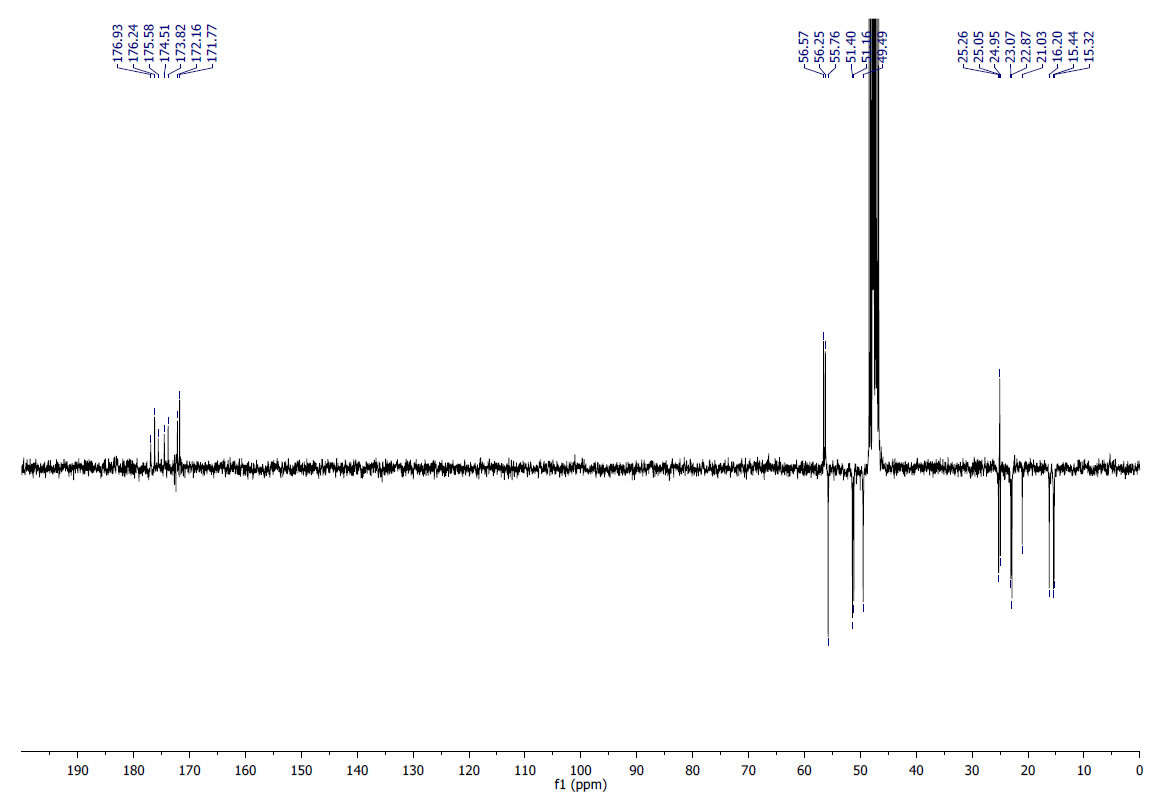
Figure S10. ^13^C-NMR of peptide 2 (75 MHz, CD_3_OD).

**Peptide 3**

Figure S11. Chemical structure of peptide 3 (HS-CH_2_CH_2_CO-A-Aib-A-Aib-A-NH_2_).


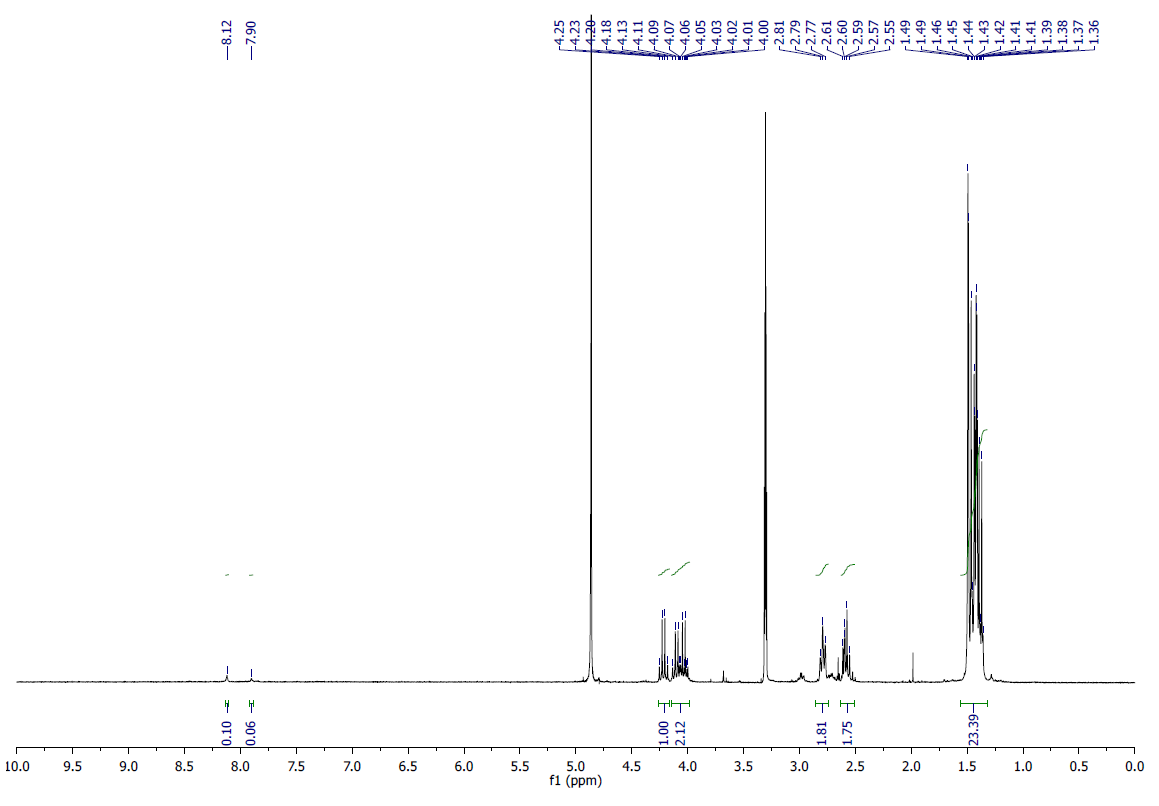
Figure S12. ^1^H-NMR of peptide 3 (300 MHz, CD_3_OD).


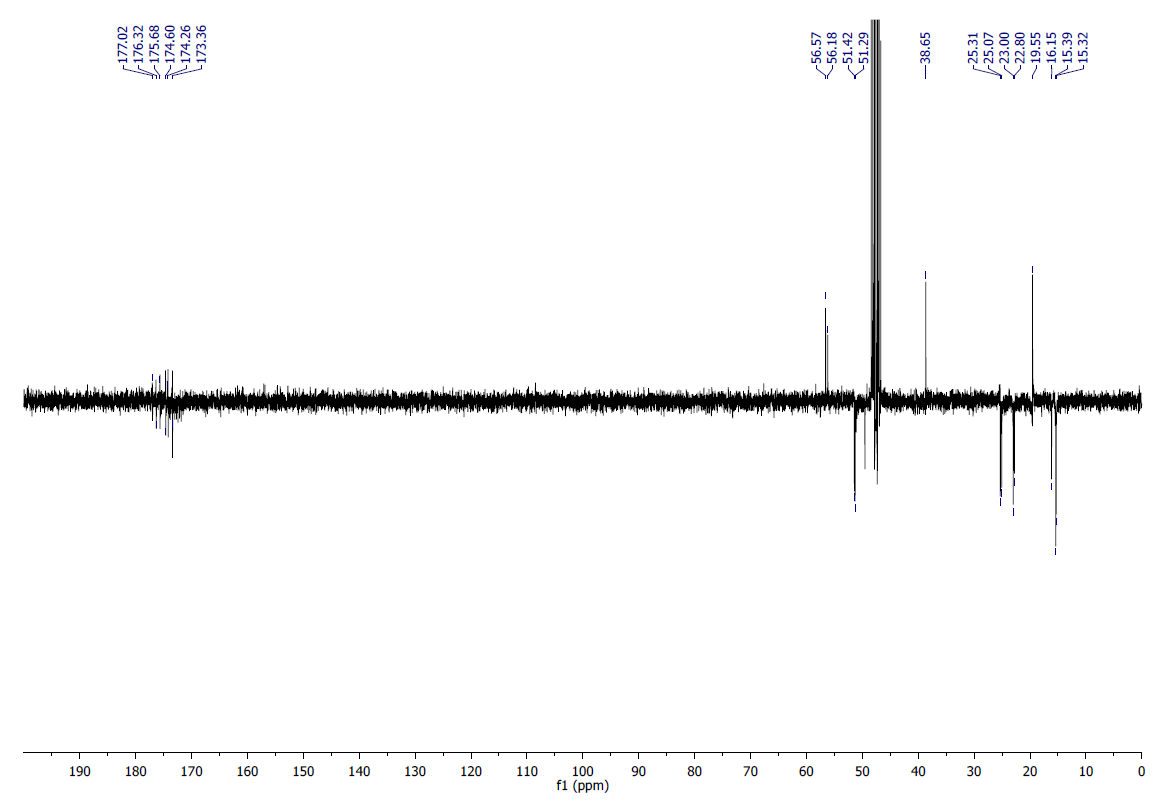
Figure S13. ^13^C-NMR of peptide 3 (75 MHz, CD_3_OD).

**Peptide 4**

Figure S14. Chemical structure of peptide 4 (Ac-A-Aib-A-C-NH_2_).


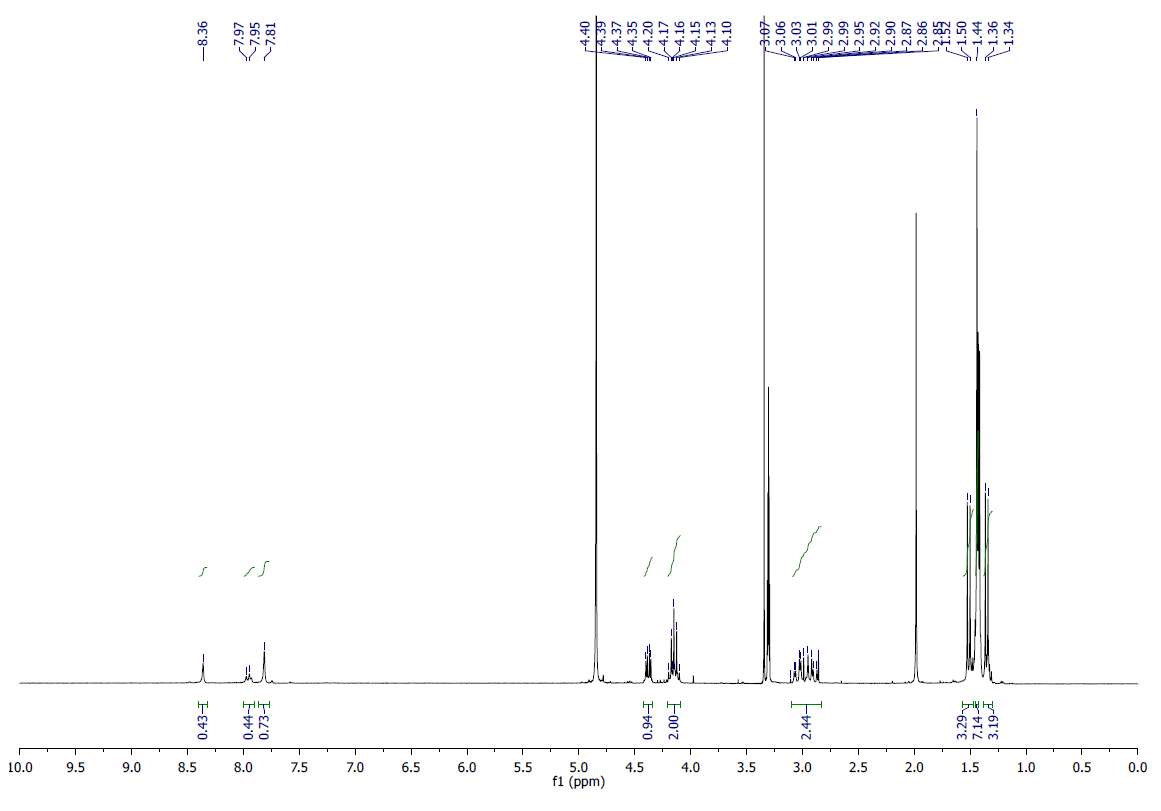
Figure S15. ^1^H-NMR of peptide 4 (300 MHz, CD_3_OD).


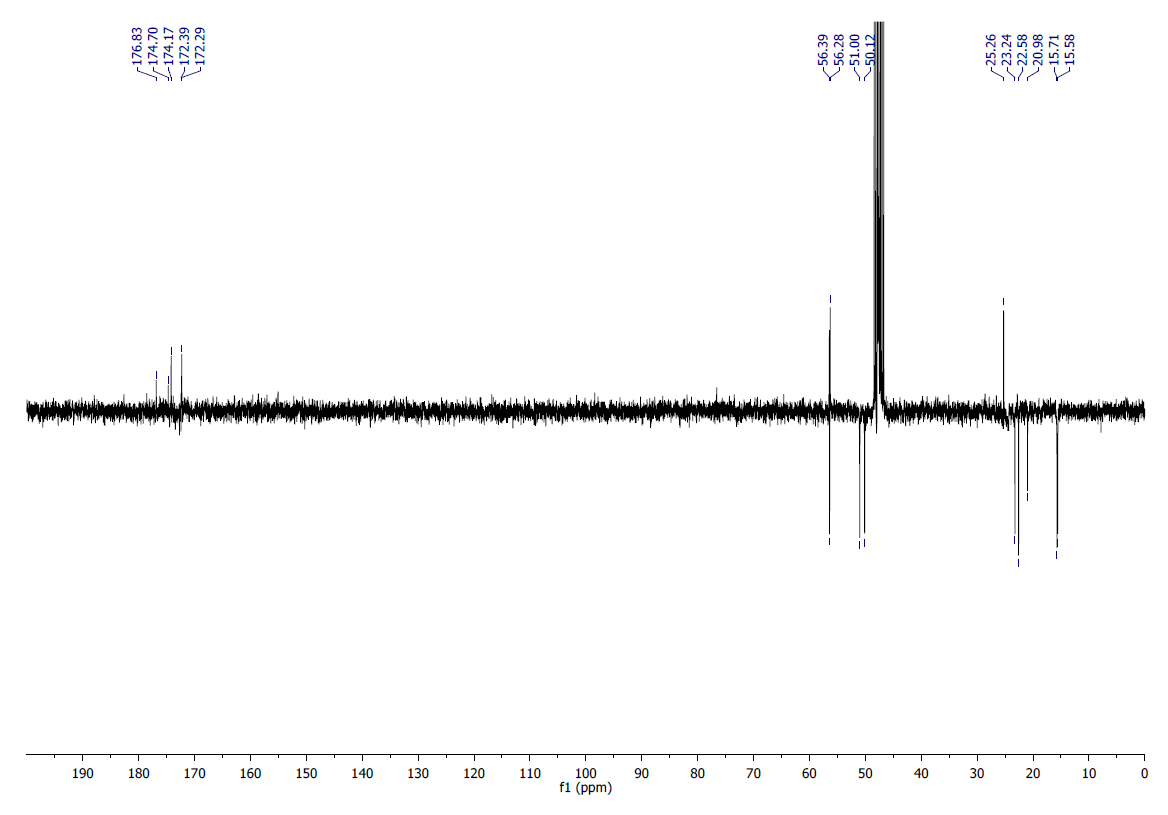
Figure S16. ^13^C-NMR of peptide 4 (75 MHz, CD_3_OD).

**Peptide 5**

Figure S17. Chemical structure of peptide 5 (HS-CH_2_CH_2_CO-A-Aib-A-NH_2_).


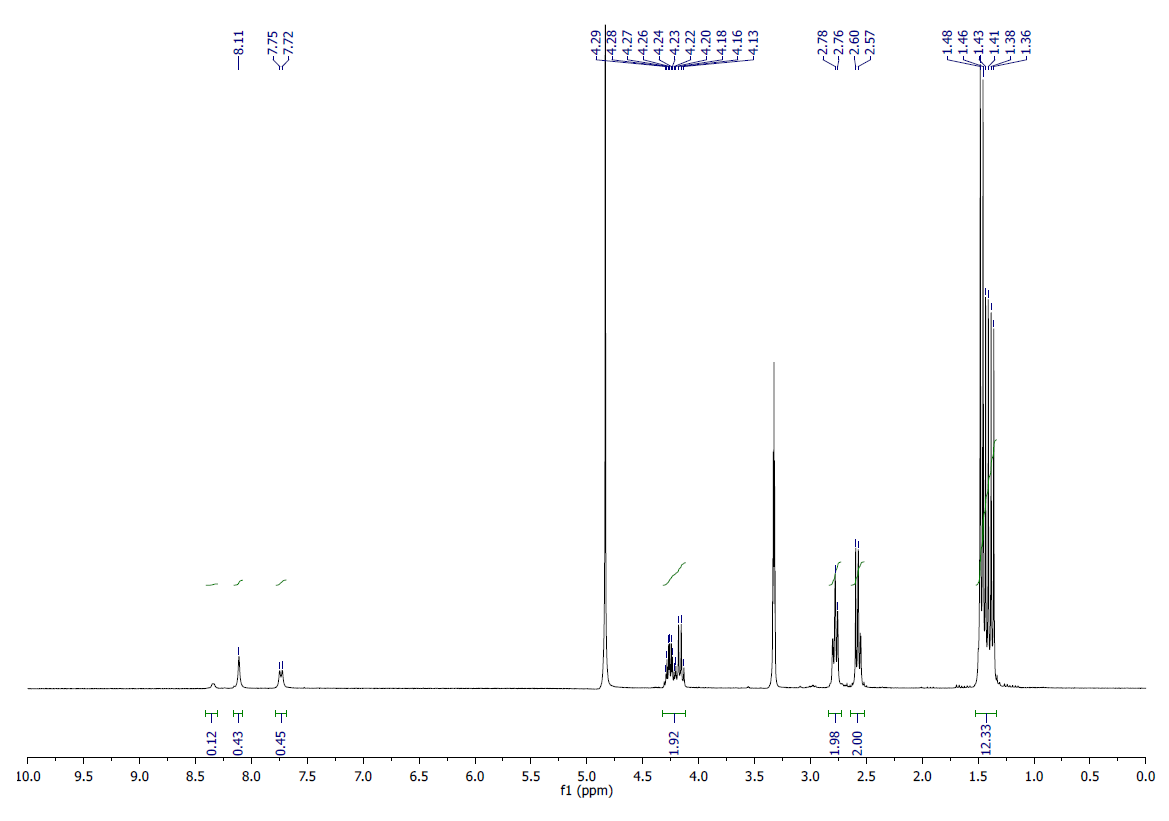
Figure S18. ^1^H-NMR of peptide 4 (300 MHz, CD_3_OD).


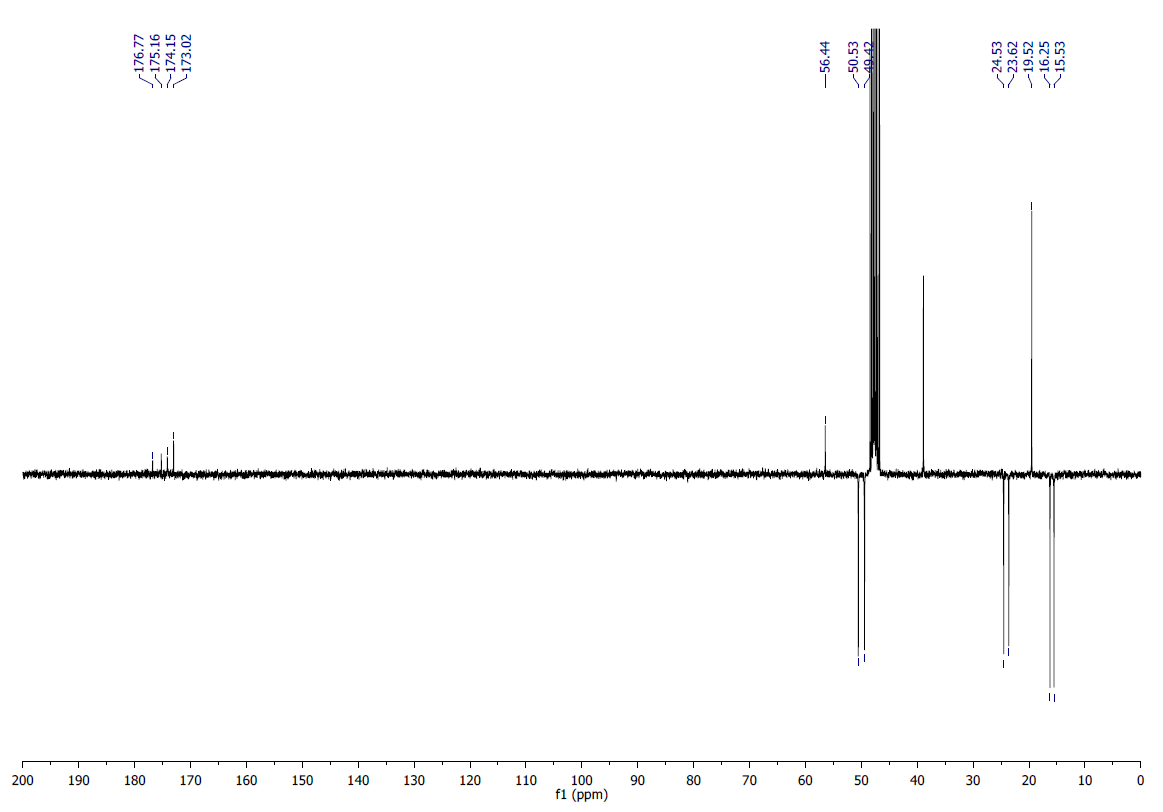
Figure S19. ^13^C-NMR of peptide 4 (75 MHz, CD_3_OD).

**Peptide 6**

Figure S20. Chemical structure of peptide 6 (Ac-A-Ac5c-A-Aib-A-C-NH_2_).


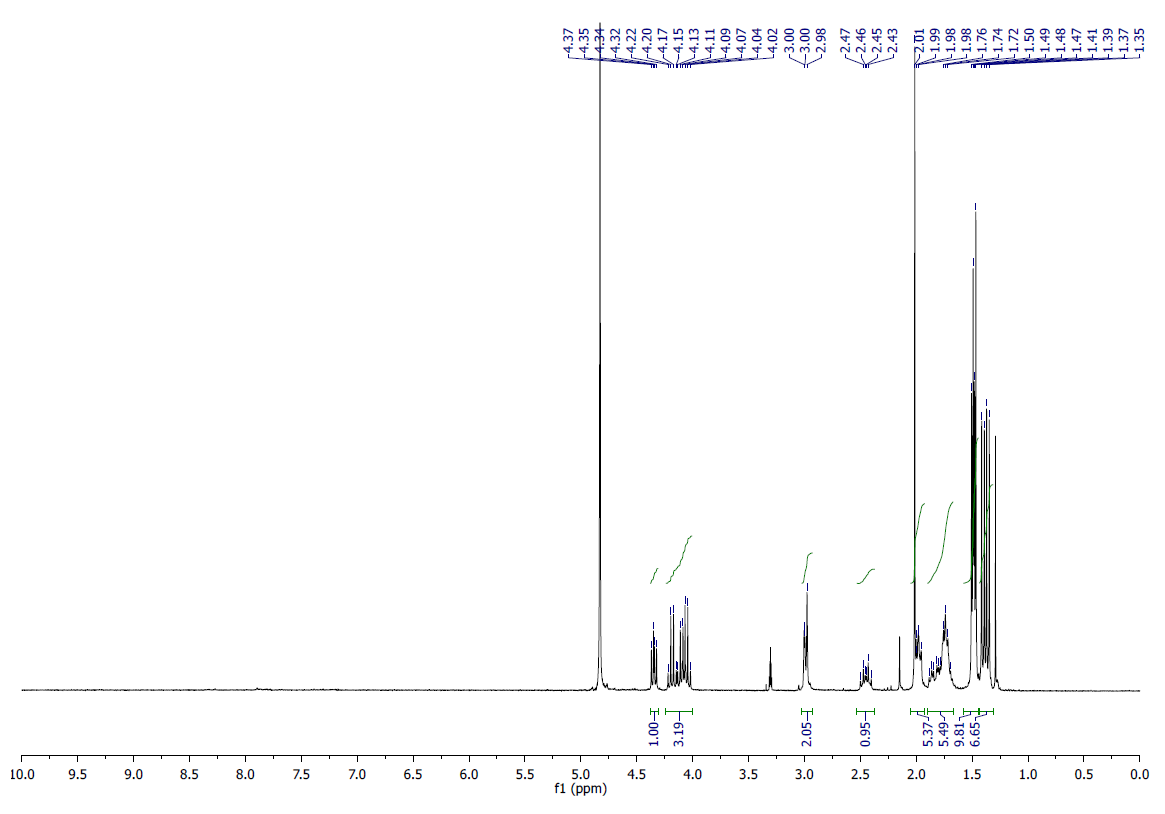
Figure S21. ^1^H-NMR of peptide 6 (300 MHz, CD_3_OD).


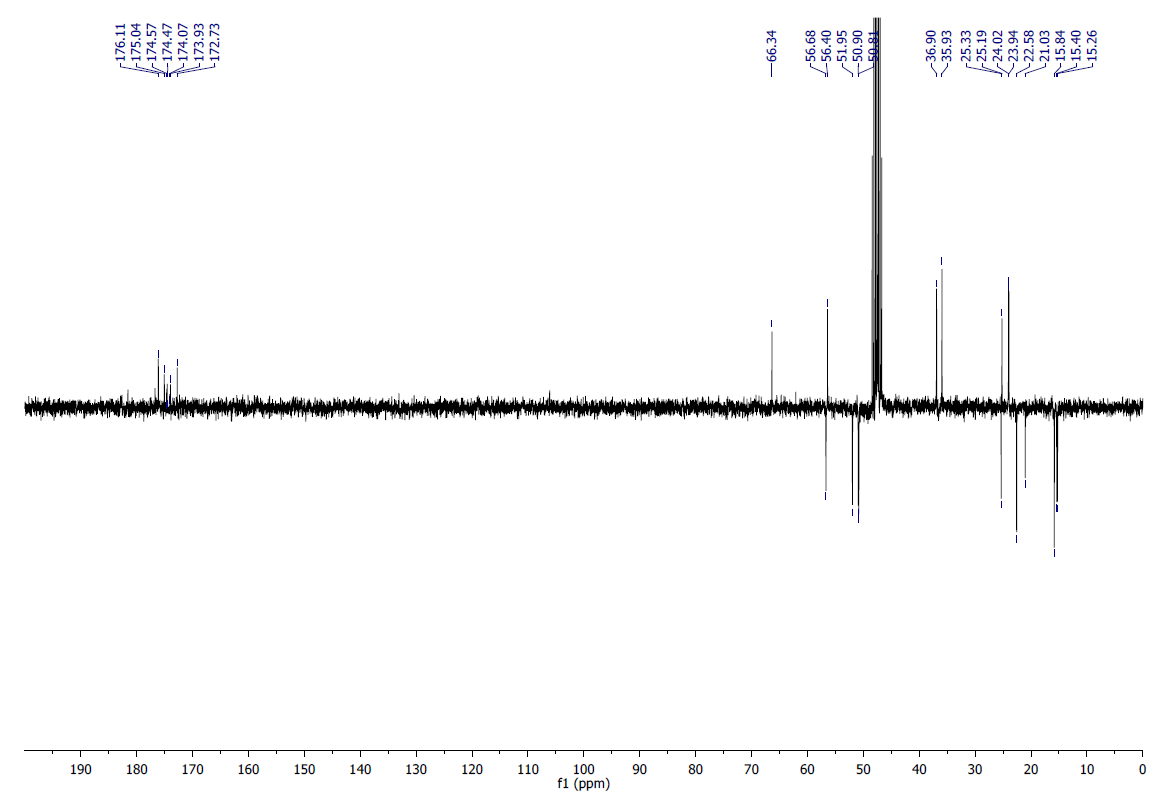
Figure S22. ^13^C-NMR of peptide 6 (75 MHz, CD_3_OD).

**Peptide 7**

Figure S23. Chemical structure of peptide 7 (HS-CH_2_CH_2_CO-A-Ac5C-A-Aib-A-NH_2_).


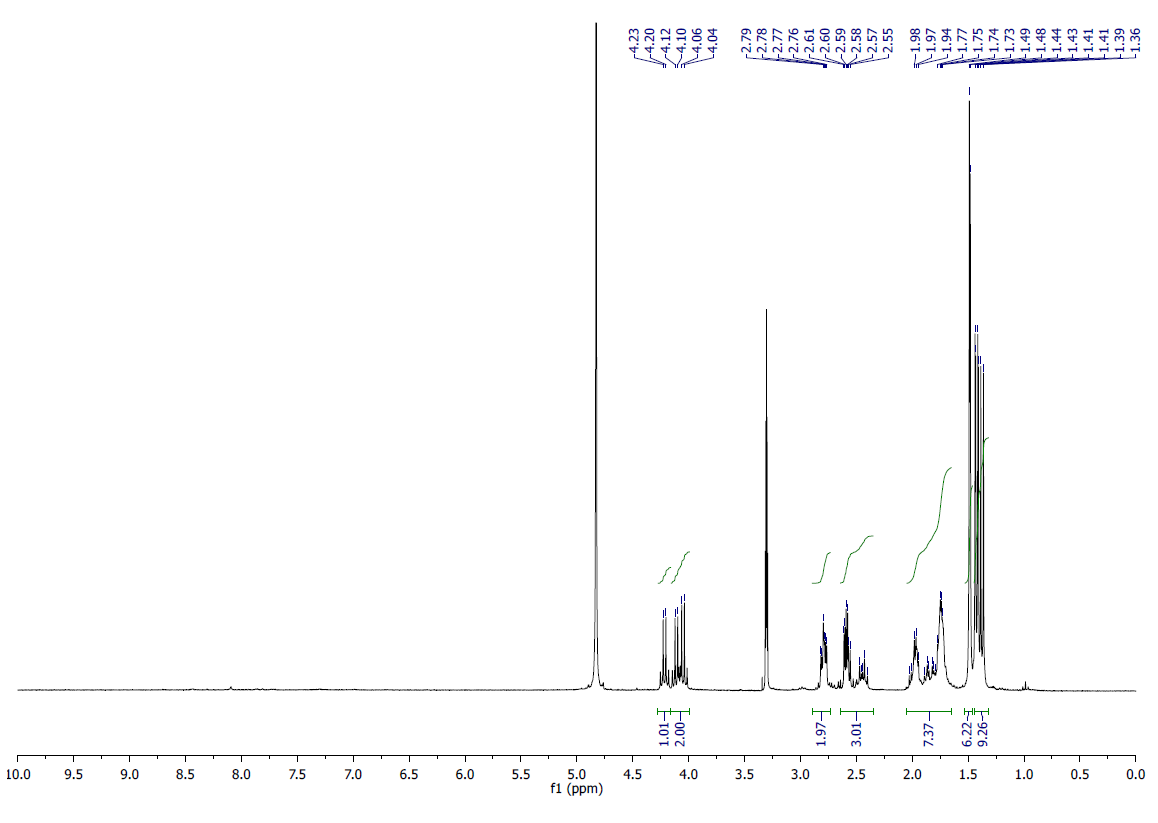
Figure S24. ^1^H-NMR of peptide 7 (300 MHz, CD_3_OD).


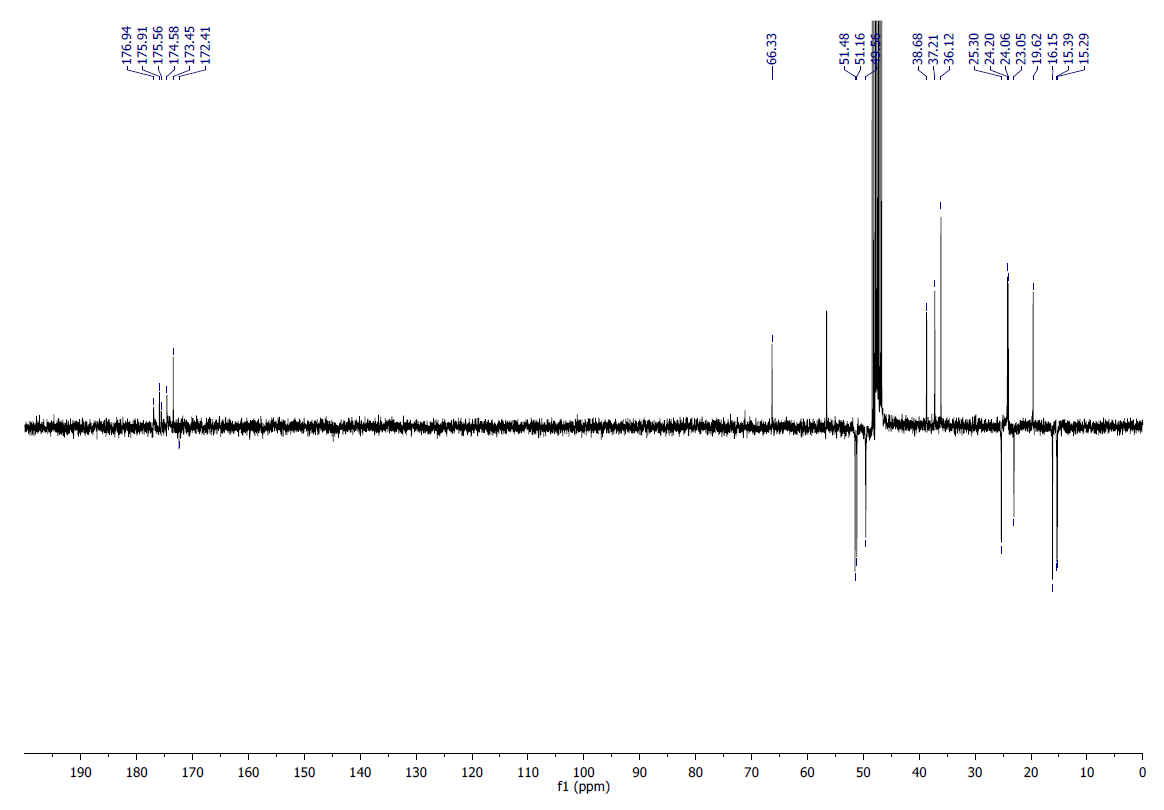
Figure S25. ^13^C-NMR of peptide 7 (75 MHz, CD_3_OD).

**Peptide 8**

Figure S26. Chemical structure of peptide 8 (HS-CH_2_CH_2_CO-A-Ac5c-A-NH_2_).


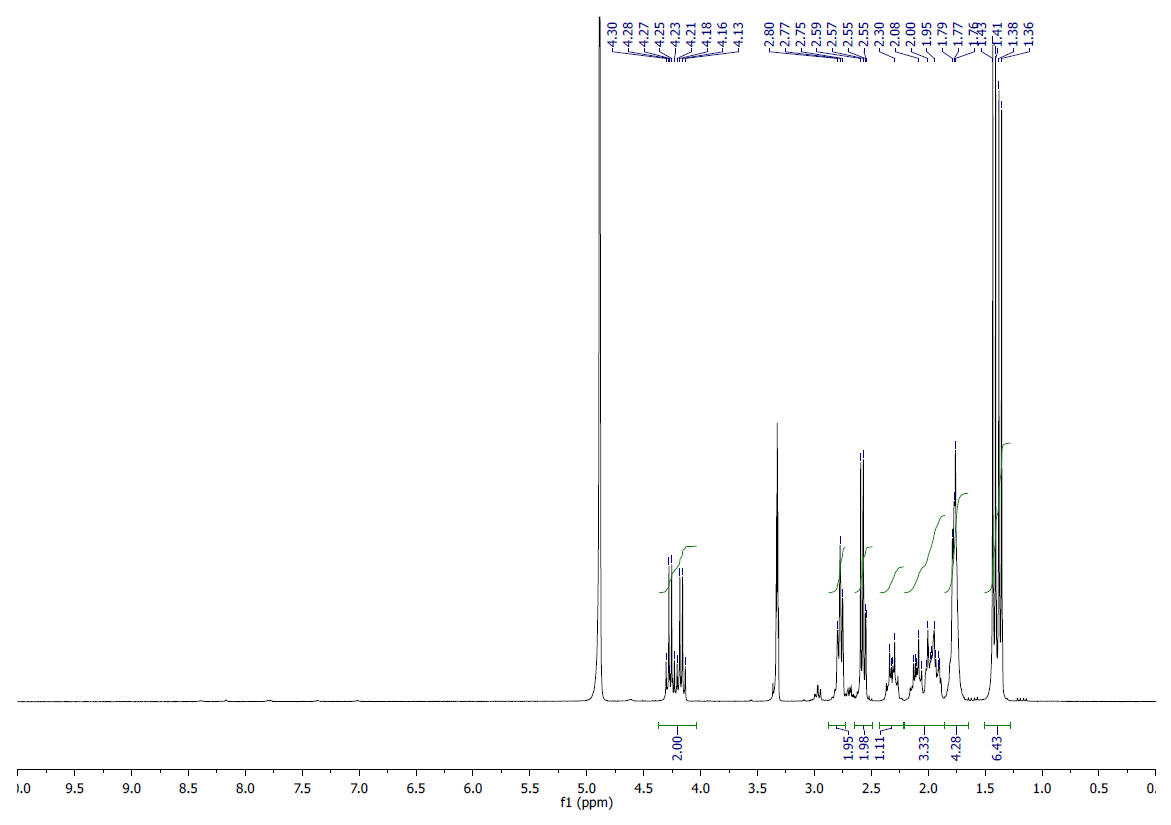


Figure S27. ^1^H-NMR of peptide 8 (300 MHz, CD_3_OD).


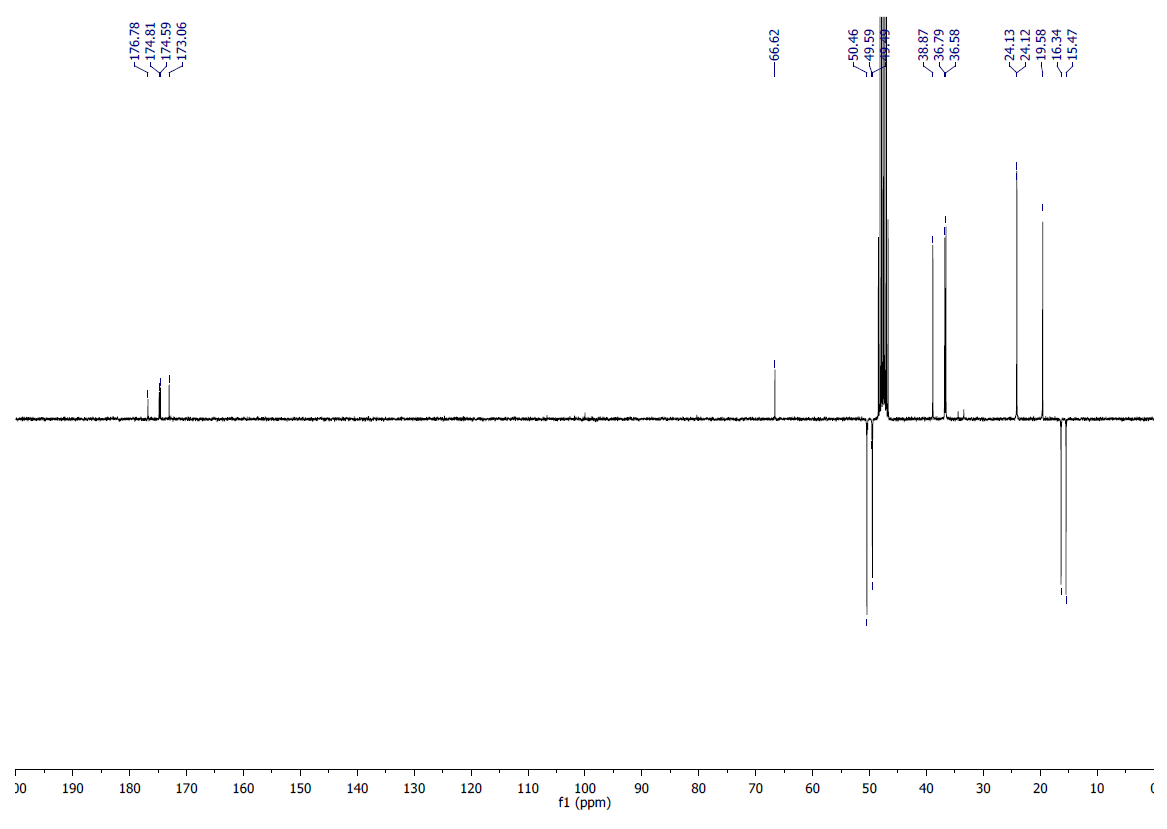


Figure S28. ^13^C-NMR of peptide 8 (75 MHz, CD_3_OD).

**Peptide 9**


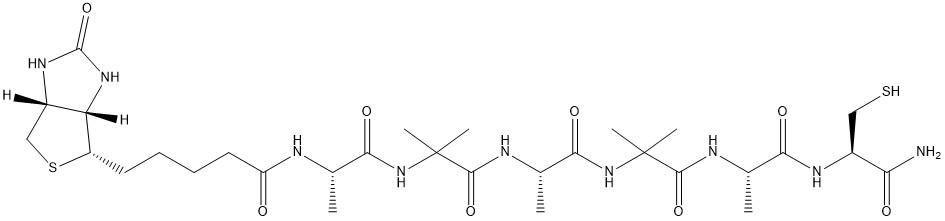


Figure S29. Chemical structure of peptide 10 (Biotin-A-Aib-A-Aib-A-C-NH_2_).


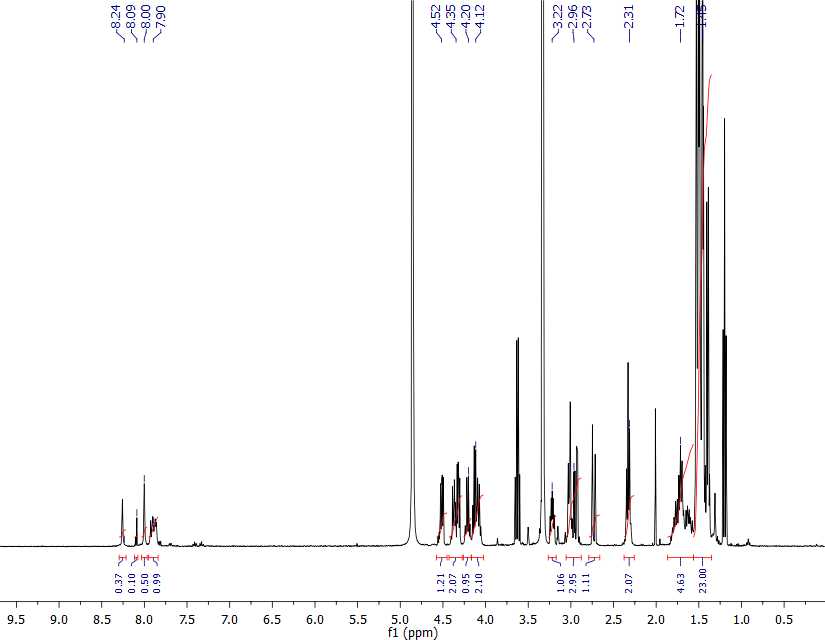


Figure S30. ^1^H-NMR of peptide 9 (400 MHz, CD_3_OD).


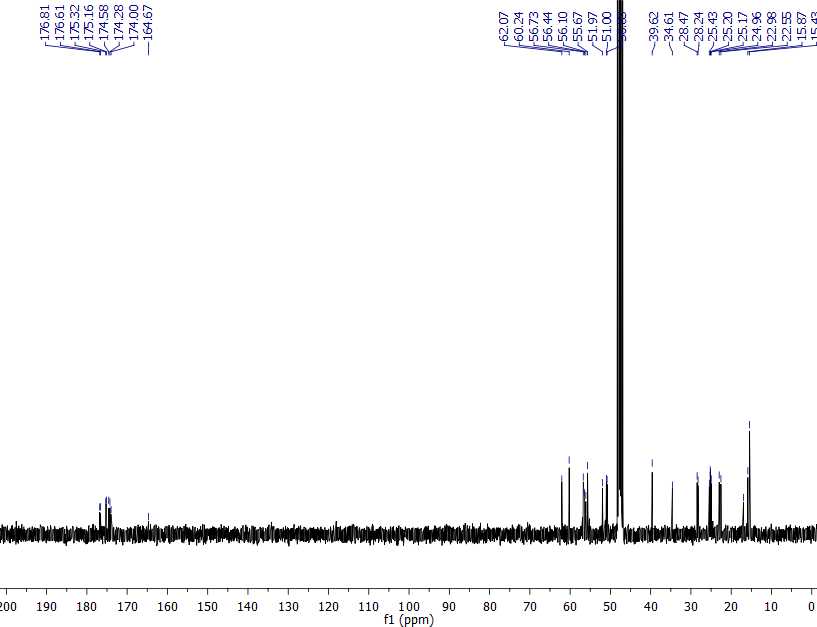


Figure S31. ^13^C-NMR of peptide 9 (100 MHz, CD_3_OD).

**Peptide 10**


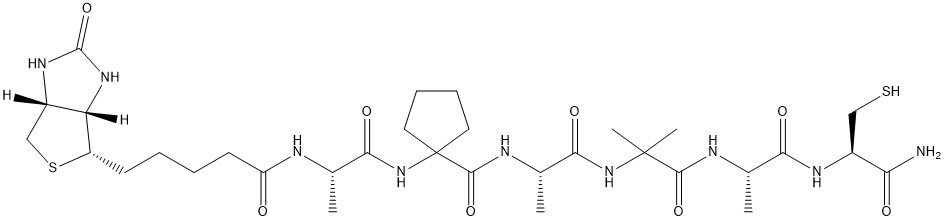


Figure S32. Chemical structure of peptide 10 (Biotin-A-Ac5c-A-Aib-A-C-NH_2_).


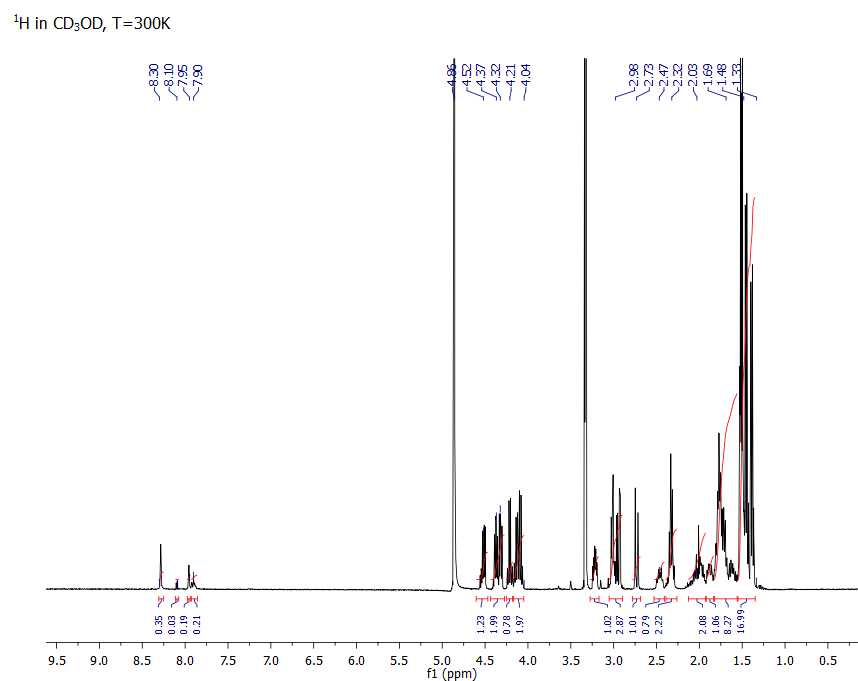


Figure S33. ^1^H-NMR of peptide 10 (400 MHz, CD_3_OD).


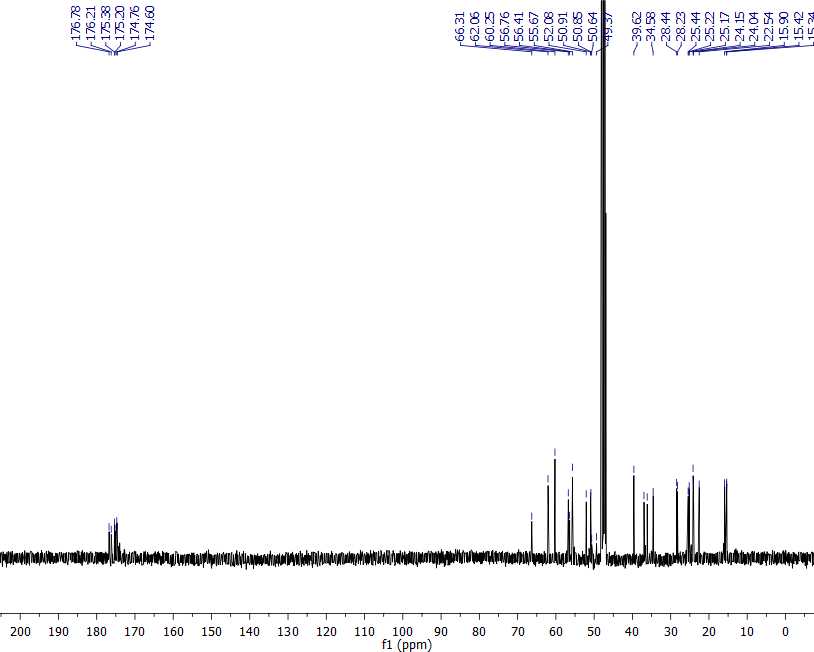


Figure S34. ^13^C-NMR of peptide 10 (100 MHz, CD_3_OD).
